# Supplementary material for: A Systemic Insight into Exohedral Actinides and Endohedral Borospherenes: An&Bm and An@Bn (An=U, Np, Pu; m = 28, 32, 34, 36, 38, 40; n = 36, 38, 40)
Source: Molecules. 2022 Sep 16;27(18):6047. doi: 10.3390/molecules27186047 (PMC9500945; doi:10.3390/molecules27186047)
Supplement: Supplementary file 1 [file molecules-27-06047-s001.zip › molecules-1881995-supplementary.pdf]

Supporting Information

A Systemic Insight of Actinide Exohedral and Endohedral Borospherenes: An&B_m and An@B_n (An=U, Np, Pu; m=28, 32, 34, 36, 38, 40; n= 36, 38, 40)

Contents:

Table S1. Cartesian coordinates for the An&B_m (An = U, Np, Pu; m=28, 32, 34, 36, 38, 40) and An@B_n (An = U, Np, Pu; n= 36, 38, 40) at PBE-ZORA/def2- TZVPP -SARC level of theory.
Table S2. Selected bond lengths [Å], fuzzy bond order (FBO) and VDD charge (e) of An&B_m (An = U, Np, Pu; m = 28, 32, 34, 36, 38, 40) clusters.
Table S3. Topological parameters for the An–B bond critical points (BCPs) of the An&B_m (An = U, Np, Pu; m = 28, 32, 34, 36, 38, 40) clusters at PBE-ZORA/def2-TZVPP level.
Table S4. Hirshfeld atomic charge of An&B_{36} and An@B_{36} (Heavy elements in bold)
Table S5. Hirshfeld atomic charge of An&B_{38} and An@B_{38} (Heavy elements in bold)
Table S6. Hirshfeld atomic charge of An&B_{40} and An@B_{40} (Heavy elements in bold)
Table S7. *f* orbital information of the U&Bm and U@Bn (m, n = 36, 38, 40) (M is Mulliken and E is Loewdin)
Table S8. *f* orbital information of the Pu&Bm and Pu @Bn (m, n = 36, 38, 40) (M is Mulliken and E is Loewdin)
Table S9. *f* orbital information of the Np&Bm and Np @Bn (m, n = 36, 38, 40) (M is Mulliken and E is Loewdin)
Table S10. The information of dipole moment for Endohedral Borospherenes
Table S11. The information of dipole moment for Exohedral Borospherenes
Figure S1. ESP-mapped molecular vdW surfaces of B_m (m = 28, 32, 34, 36, 38, 40) at the PBE/def2-TZVPP level.
Figure S2. The TDOS, PDOS and OPDOS curves of Pu&B_{36} at the PBE-ZORA/def2-TZVPP-SARC level.
Figure S3. IR spectra of An&B_m (An = U, Np, Pu; m = 28, 32, 34, 38, 40) at PBE-ZORA/def2-TZVPP-SARC level.
Figure S4. UV/Vis spectra of the An&B_m (An = U, Np, Pu; m = 28, 32, 34 38, 40) in vacuum (solid lines) and in water using the CPCM (dash lines) at the PBE0-ZORA/def2-TZVPP-SARC level.
Figure S5. IR spectra of An@B_n (An = U, Np, Pu; n = 36, 38) at the PBE-ZORA/def2-TZVPP-SARC level.
Figure S6. UV/Vis spectra of the An@B_n (An = U, Np, Pu; n = 36, 38, 40) in vacuum (solid lines) and in water using the CPCM (dash lines) at the PBE0-ZORA/def2-TZVPP-SARC level.
Figure S7. QTAIM molecular graph of Np@Bn and Pu@Bn (n=36，38，40). Red points represent bond critical points, lines represent bond paths.
Figure S8. Two-dimensional color-filled plane map of ELF for Np@Bn and Pu@Bn (n = 36, 38,

40) at the PBE-ZORA/def2-SVP-SARC level.

Figure S9. Charge-transfer spectrum (CTS) of AnB_n (An = U, Np, Pu; n = 36, 38, 40) at PBE0 ZORA/def2-TZVPP-SARC level.

Table S1. Cartesian coordinates for the An&B_m (An = U, Np, Pu; m=28, 32, 34, 36, 38, 40) and An@B_n (An = U, Np, Pu; n= 36, 38, 40) at PBE-ZORA/def2-TZVPP-SARC level of theory.

U&B_{28}

Np	2.492897000	1.500238000	-1.570771000
B	-0.174215000	2.056548000	-1.346474000
B	0.378149000	-0.541913000	1.921728000
B	0.908065000	-2.058573000	1.341849000
B	-1.349953000	-0.299243000	2.069480000
B	0.748979000	-1.850216000	-1.578561000
B	-0.339633000	0.964716000	2.249883000
B	1.261407000	0.907468000	1.743951000
B	1.954565000	2.181627000	0.773914000
B	2.294991000	-1.141920000	-1.525596000
B	-2.321312000	-1.495378000	1.526430000
B	-1.370043000	0.787474000	-1.777447000
B	1.938704000	-0.733337000	1.256150000
B	-1.695823000	-1.932877000	0.053586000
B	-2.742272000	-0.535122000	0.288324000
B	2.919515000	-0.518057000	-0.136820000
B	-0.466072000	-0.692796000	-2.137989000
B	-0.874444000	-2.134375000	-1.362554000
B	1.183309000	-0.412178000	-2.458524000
B	-1.890509000	-0.781313000	-1.215970000
B	-0.786844000	2.891697000	0.056296000
B	1.739532000	-1.817189000	-0.078643000
B	-2.796860000	0.630847000	-0.871184000
B	0.158376000	0.891088000	-2.502740000
B	0.214493000	2.193231000	1.264147000
B	-1.877369000	1.933572000	-0.587114000
B	2.753108000	0.762385000	0.849698000
B	-0.715192000	-1.879937000	1.571922000
B	0.818142000	2.873118000	-0.193641000

Electronic energy = -30108.79624990 (Eh)
Gibbs free enthalpy = -30108.71141520 (Eh)
Enthalpy = -30108.65066632 (Eh)

Np&B_{28}

Np	2.519961000	1.512797000	-1.586847000
B	-0.165724000	2.065978000	-1.347807000
B	0.366619000	-0.545037000	1.916049000
B	0.905349000	-2.065760000	1.339792000
B	-1.364333000	-0.310834000	2.043908000
B	0.751845000	-1.861388000	-1.579931000
B	-0.362609000	0.961725000	2.214847000
B	1.239494000	0.902058000	1.717202000
B	1.941893000	2.185971000	0.772097000

B	2.300028000	-1.165573000	-1.527946000
B	-2.322380000	-1.534527000	1.534260000
B	-1.351831000	0.789529000	-1.753588000
B	1.928131000	-0.729034000	1.247487000
B	-1.699184000	-1.949006000	0.055343000
B	-2.722633000	-0.540562000	0.316923000
B	2.904750000	-0.517536000	-0.140852000
B	-0.460289000	-0.687724000	-2.126444000
B	-0.872071000	-2.136667000	-1.357482000
B	1.186773000	-0.414374000	-2.439679000
B	-1.883224000	-0.778896000	-1.200771000
B	-0.784028000	2.932431000	0.038932000
B	1.739293000	-1.828582000	-0.078269000
B	-2.770824000	0.639606000	-0.829018000
B	0.167991000	0.888560000	-2.489132000
B	0.204240000	2.205090000	1.250895000
B	-1.872267000	1.965122000	-0.594654000
B	2.739170000	0.769593000	0.838817000
B	-0.716177000	-1.899971000	1.583952000
B	0.815726000	2.896593000	-0.194757000

Electronic energy = -30978.98688422 (Eh)
Gibbs free enthalpy = -30978.89852054 (Eh)
Enthalpy = -30978.83853615 (Eh)

Pu&B_{28}

Pu	2.515453000	1.502226000	-1.592193000
B	-0.144428000	2.071960000	-1.356981000
B	0.347528000	-0.556007000	1.910882000
B	0.890644000	-2.082434000	1.352529000
B	-1.394960000	-0.315857000	2.000396000
B	0.740373000	-1.876735000	-1.568164000
B	-0.386663000	0.950175000	2.184686000
B	1.225104000	0.886204000	1.715900000
B	1.936013000	2.181938000	0.799027000
B	2.293740000	-1.195627000	-1.521923000
B	-2.328399000	-1.582664000	1.544228000
B	-1.342009000	0.785136000	-1.739342000
B	1.913656000	-0.740499000	1.249272000
B	-1.711017000	-1.972871000	0.060930000
B	-2.688276000	-0.530428000	0.364173000
B	2.888599000	-0.539687000	-0.135755000
B	-0.463302000	-0.700683000	-2.126051000
B	-0.891775000	-2.145108000	-1.356235000
B	1.180635000	-0.438858000	-2.430266000
B	-1.878741000	-0.778838000	-1.184920000
B	-0.764089000	2.967628000	0.016088000

B	1.726453000	-1.850159000	-0.068050000
B	-2.737883000	0.642380000	-0.789219000
B	0.180794000	0.875680000	-2.479326000
B	0.205186000	2.204996000	1.249743000
B	-1.840061000	1.983887000	-0.608176000
B	2.719118000	0.751850000	0.836391000
B	-0.725317000	-1.928860000	1.601262000
B	0.832717000	2.905440000	-0.176374000

Electronic energy = -31868.67490697 (Eh)
Gibbs free enthalpy = -30108.71141520 (Eh)
Enthalpy = -31868.59836356 (Eh)

U&B_{32}

U	2.258318000	0.159887000	2.921589000
B	-1.231364000	1.619722000	2.292517000
B	-1.875511000	-1.687156000	0.940558000
B	1.284353000	-2.437437000	-0.514427000
B	-0.146218000	0.683606000	3.095227000
B	2.026854000	0.237479000	0.165831000
B	-1.605304000	-0.380276000	-1.658379000
B	-0.479804000	-0.587105000	-2.956360000
B	-0.855889000	2.801006000	1.203391000
B	-0.387457000	3.785671000	-0.053297000
B	0.818502000	2.802512000	0.504636000
B	0.369380000	-3.830834000	-0.209352000
B	1.932500000	1.683368000	1.048783000
B	1.808927000	-1.359719000	0.794591000
B	1.708902000	0.409185000	-1.607318000
B	1.149661000	-1.982934000	2.332106000
B	-0.572230000	-1.902948000	1.920098000
B	1.636600000	1.792863000	-0.607978000
B	1.176007000	1.905912000	-2.229612000
B	0.118908000	-0.971054000	3.118467000
B	0.461340000	1.762321000	1.979145000
B	-1.793160000	1.305114000	0.730659000
B	-1.162073000	-1.879156000	-2.295022000
B	-0.831031000	-2.831935000	0.430173000
B	0.349237000	2.934957000	-1.226968000
B	-1.663392000	-1.801277000	-0.705289000
B	-1.968483000	1.067580000	-0.921297000
B	-1.306438000	2.468834000	-0.515080000
B	-2.024332000	-0.250711000	0.099158000
B	2.015426000	-1.056717000	-0.877756000
B	0.592633000	0.562883000	-2.903375000
B	-0.286559000	-2.911860000	-1.339127000
B	0.880241000	-3.011776000	1.110823000

Electronic energy = -30207.94543484 (Eh)
Gibbs free enthalpy = -30207.85777730 (Eh)
Enthalpy = -30207.78652380 (Eh)

Np&B_{32}

Np	2.271278000	0.161466000	2.931035000
B	-1.239041000	1.608725000	2.281528000
B	-1.882857000	-1.688721000	0.929492000
B	1.276591000	-2.441124000	-0.504417000
B	-0.144832000	0.694630000	3.107404000
B	2.011510000	0.233065000	0.197217000
B	-1.617139000	-0.383900000	-1.673543000
B	-0.478043000	-0.577939000	-2.959628000
B	-0.871245000	2.802423000	1.206495000
B	-0.388752000	3.778712000	-0.046230000
B	0.819859000	2.811602000	0.529432000
B	0.363171000	-3.838601000	-0.209532000
B	1.921494000	1.689918000	1.056550000
B	1.795384000	-1.370251000	0.811311000
B	1.697742000	0.405184000	-1.586474000
B	1.123229000	-1.990046000	2.344657000
B	-0.578223000	-1.917299000	1.903334000
B	1.631082000	1.788975000	-0.592056000
B	1.186028000	1.901328000	-2.209185000
B	0.091666000	-0.953382000	3.088143000
B	0.467857000	1.776661000	2.002705000
B	-1.796163000	1.296460000	0.720496000
B	-1.146381000	-1.878385000	-2.304418000
B	-0.843666000	-2.838913000	0.419326000
B	0.340293000	2.930543000	-1.221151000
B	-1.658913000	-1.800346000	-0.715264000
B	-1.977227000	1.064235000	-0.931790000
B	-1.301666000	2.457562000	-0.520444000
B	-2.034350000	-0.255645000	0.084834000
B	2.005768000	-1.058880000	-0.854761000
B	0.598586000	0.568915000	-2.899093000
B	-0.285322000	-2.921932000	-1.345534000
B	0.857528000	-3.021336000	1.120275000

Electronic energy = -31078.15059756 (Eh)
Gibbs free enthalpy = -31078.05339211 (Eh)
Enthalpy = -31077.98604052 (Eh)

Pu&B_{32}

Pu	2.296089000	0.125762000	2.880816000
B	-1.243965000	1.625327000	2.283499000
B	-1.889918000	-1.691189000	0.914487000

B	1.252623000	-2.454556000	-0.546497000
B	-0.193005000	0.633408000	3.035323000
B	1.999107000	0.213873000	0.163329000
B	-1.633490000	-0.386858000	-1.681485000
B	-0.512564000	-0.573753000	-2.979566000
B	-0.869836000	2.799434000	1.189461000
B	-0.381075000	3.778445000	-0.057401000
B	0.823117000	2.796173000	0.509585000
B	0.336013000	-3.849572000	-0.244030000
B	1.921478000	1.669738000	1.033452000
B	1.781814000	-1.391373000	0.768496000
B	1.672827000	0.399392000	-1.614022000
B	1.120526000	-2.019727000	2.316574000
B	-0.583295000	-1.926900000	1.885891000
B	1.627382000	1.780024000	-0.618891000
B	1.177770000	1.897291000	-2.232892000
B	0.101015000	-0.998195000	3.087525000
B	0.454473000	1.734511000	1.950601000
B	-1.792007000	1.300738000	0.711653000
B	-1.175744000	-1.878237000	-2.325538000
B	-0.859100000	-2.844793000	0.395968000
B	0.326115000	2.920957000	-1.238982000
B	-1.680132000	-1.802824000	-0.730748000
B	-1.973131000	1.066212000	-0.941607000
B	-1.306978000	2.461137000	-0.530048000
B	-2.038494000	-0.255721000	0.073383000
B	1.979694000	-1.069336000	-0.896039000
B	0.562966000	0.578064000	-2.923405000
B	-0.314227000	-2.929963000	-1.376504000
B	0.853196000	-3.033785000	1.078328000

Electronic energy = -31967.83965344 (Eh)

Gibbs free enthalpy = -31967.75323209 (Eh)

Enthalpy = -31967.68102367 (Eh)

U&B_{34}

U	1.998974000	-1.355444000	2.904444000
B	1.147760000	2.366285000	1.364168000
B	-2.260819000	0.978381000	-1.018511000
B	0.850071000	-1.445720000	-1.797295000
B	2.381764000	-0.201087000	0.685987000
B	2.329302000	-1.001013000	-0.908827000
B	-0.474675000	-1.538621000	2.621099000
B	-1.653943000	0.038814000	-2.328990000
B	2.235913000	0.729796000	-0.772973000
B	-0.759773000	1.462576000	-1.910889000
B	0.068190000	-0.023346000	-2.401490000

B	-0.116453000	1.710788000	2.373102000
B	-1.494116000	2.749606000	1.064980000
B	0.116955000	2.766340000	-1.402900000
B	-0.020284000	3.225397000	0.177394000
B	0.984271000	1.382576000	-1.797245000
B	1.534468000	-2.985386000	1.061575000
B	2.336848000	1.455895000	0.713301000
B	2.659835000	-1.870349000	0.507072000
B	0.036982000	-3.251016000	0.249100000
B	-0.152664000	3.260111000	1.816709000
B	-1.341082000	2.368840000	-0.613526000
B	-1.306123000	-2.326924000	1.322809000
B	-2.536396000	0.188606000	0.520041000
B	1.208183000	0.657070000	1.745813000
B	-2.255492000	-0.726420000	-0.960655000
B	-2.651681000	1.860463000	0.355850000
B	-0.088129000	-2.760536000	-1.330899000
B	1.776449000	-0.031834000	-2.218954000
B	-1.495941000	-0.643553000	1.633869000
B	-0.223747000	0.104265000	2.629942000
B	1.335419000	-2.394789000	-0.524631000
B	-2.409963000	-1.481280000	0.486580000
B	0.150273000	-2.900536000	1.910862000
B	-0.887122000	-1.382900000	-1.823595000

Electronic energy = -30257.62528431 (Eh)
Gibbs free energy = -30257.50080901 (Eh)
Enthalpy = -30257.43786675 (Eh)

Np&B_{34}

Np	2.117290000	-1.388710000	2.793821000
B	1.227819000	2.352774000	1.302309000
B	-2.269721000	0.979032000	-0.961382000
B	0.814596000	-1.436748000	-1.875103000
B	2.436957000	-0.214653000	0.576402000
B	2.320765000	-0.995190000	-1.032934000
B	-0.372266000	-1.570213000	2.582713000
B	-1.707906000	0.048787000	-2.305791000
B	2.217307000	0.736738000	-0.869800000
B	-0.806404000	1.472088000	-1.911424000
B	0.006904000	-0.005457000	-2.441736000
B	-0.015168000	1.680882000	2.328569000
B	-1.425631000	2.732358000	1.105229000
B	0.088028000	2.773339000	-1.427674000
B	0.005286000	3.222812000	0.156711000
B	0.941486000	1.396900000	-1.871813000
B	1.599425000	-3.012655000	0.935713000

B	2.400692000	1.441944000	0.618207000
B	2.698471000	-1.882249000	0.351666000
B	0.071758000	-3.262192000	0.182426000
B	-0.055541000	3.242449000	1.799038000
B	-1.337464000	2.363675000	-0.583864000
B	-1.236240000	-2.353844000	1.307115000
B	-2.507517000	0.164118000	0.569066000
B	1.271680000	0.634727000	1.642879000
B	-2.278100000	-0.731810000	-0.934283000
B	-2.608847000	1.840573000	0.435445000
B	-0.111555000	-2.746821000	-1.385300000
B	1.718204000	-0.014319000	-2.309873000
B	-1.421068000	-0.674359000	1.632873000
B	-0.109784000	0.073770000	2.574639000
B	1.339063000	-2.393003000	-0.626072000
B	-2.371838000	-1.501482000	0.508534000
B	0.247135000	-2.917220000	1.841036000
B	-0.932844000	-1.375401000	-1.842348000

Electronic energy = -31127.81814566 (Eh)
Gibbs free enthalpy = -31127.70140562 (Eh)
Enthalpy = -31127.63599853 (Eh)

Pu&B_{34}

Pu	2.120669000	-1.396384000	2.808288000
B	1.229107000	2.348129000	1.300283000
B	-2.274898000	0.982013000	-0.964265000
B	0.812200000	-1.438469000	-1.877614000
B	2.448891000	-0.221414000	0.573947000
B	2.325961000	-0.992548000	-1.034477000
B	-0.364775000	-1.569698000	2.578368000
B	-1.710270000	0.050826000	-2.306983000
B	2.205005000	0.736989000	-0.861188000
B	-0.808697000	1.472616000	-1.912078000
B	0.004506000	-0.003999000	-2.445533000
B	-0.016459000	1.676158000	2.326640000
B	-1.432003000	2.739727000	1.102390000
B	0.087591000	2.773835000	-1.427132000
B	0.002716000	3.228072000	0.153366000
B	0.934967000	1.394371000	-1.864932000
B	1.605281000	-3.002276000	0.936546000
B	2.407429000	1.431426000	0.637251000
B	2.702527000	-1.887194000	0.341555000
B	0.062292000	-3.243015000	0.189708000
B	-0.056766000	3.236205000	1.794376000
B	-1.339454000	2.364425000	-0.583779000
B	-1.230368000	-2.351848000	1.307701000

B	-2.505166000	0.165820000	0.569617000
B	1.247216000	0.625931000	1.619467000
B	-2.278334000	-0.731190000	-0.934226000
B	-2.610914000	1.842321000	0.433484000
B	-0.112309000	-2.744146000	-1.384003000
B	1.715285000	-0.018929000	-2.304394000
B	-1.408800000	-0.668156000	1.624799000
B	-0.101620000	0.072901000	2.578450000
B	1.344412000	-2.397507000	-0.627697000
B	-2.370315000	-1.498568000	0.510422000
B	0.253658000	-2.922872000	1.846424000
B	-0.933592000	-1.372909000	-1.839788000

Electronic energy = -32017.52306392 (Eh)
Gibbs free energy = -32017.42241183 (Eh)
Enthalpy = -32017.35143020 (Eh)

U&B_{36}

U	-4.287395000	-0.005153000	0.017191000
B	-2.694428000	-0.009879000	1.757471000
B	2.685843000	0.007414000	-1.706297000
B	1.685645000	2.646796000	-0.001550000
B	0.009196000	1.678810000	2.645698000
B	-1.369607000	0.855218000	-2.290028000
B	-2.715236000	-0.019290000	-1.737796000
B	2.322080000	1.392899000	-0.887396000
B	-2.336388000	1.388742000	0.902995000
B	2.312835000	1.387814000	0.892316000
B	1.421938000	0.872794000	-2.325457000
B	-1.371270000	0.881762000	2.310086000
B	1.425072000	0.883515000	2.325685000
B	2.678839000	-0.000154000	1.695294000
B	0.887824000	2.275584000	1.369291000
B	0.001355000	1.661746000	-2.634030000
B	-2.342471000	1.385567000	-0.893868000
B	-0.917323000	2.310835000	1.426678000
B	0.876508000	2.269997000	-1.366316000
B	-1.737323000	2.651501000	0.001841000
B	-0.923384000	2.296227000	-1.410946000
B	1.713218000	-2.645217000	-0.003301000
B	0.012836000	-1.671869000	-2.632953000
B	-1.330811000	-0.867477000	2.281649000
B	2.337027000	-1.392964000	0.897719000
B	-2.343752000	-1.425514000	-0.894014000
B	2.335019000	-1.381997000	-0.891807000
B	1.417626000	-0.880845000	2.317785000
B	-1.376292000	-0.895149000	-2.296093000

B	1.432473000	-0.877525000	-2.325735000
B	0.903288000	-2.267799000	-1.368354000
B	0.020780000	-1.701764000	2.642522000
B	-2.325704000	-1.403300000	0.892898000
B	-0.903816000	-2.315045000	-1.408191000
B	0.906394000	-2.284603000	1.363442000
B	-1.720029000	-2.675464000	0.005295000
B	-0.894699000	-2.326505000	1.418334000

Electronic energy = -30307.20903135 (Eh)

Gibbs free enthalpy = -30307.03217014 (Eh)

Enthalpy = -30306.97771040 (Eh)

Np&B_{36}

Np	-4.343141000	-0.022710000	-0.004226000
B	-2.718290000	-0.007227000	1.751807000
B	2.695563000	0.002675000	-1.688087000
B	1.731149000	2.649971000	0.000369000
B	0.009694000	1.674043000	2.607714000
B	-1.356578000	0.858501000	-2.266915000
B	-2.716016000	-0.007383000	-1.758795000
B	2.358561000	1.397317000	-0.887768000
B	-2.361105000	1.389623000	0.889495000
B	2.357346000	1.397033000	0.889725000
B	1.432670000	0.870586000	-2.307230000
B	-1.359985000	0.858463000	2.262294000
B	1.429307000	0.870650000	2.307719000
B	2.693313000	0.002655000	1.690896000
B	0.908195000	2.277541000	1.358466000
B	0.013553000	1.674305000	-2.609572000
B	-2.359984000	1.389426000	-0.895960000
B	-0.903904000	2.289937000	1.373154000
B	0.910102000	2.277209000	-1.359095000
B	-1.769665000	2.662942000	-0.002548000
B	-0.902170000	2.290595000	-1.376461000
B	1.709297000	-2.644545000	0.000294000
B	0.016882000	-1.662201000	-2.619538000
B	-1.385892000	-0.880403000	2.294067000
B	2.343479000	-1.390922000	0.882664000
B	-2.345895000	-1.397981000	-0.904286000
B	2.344737000	-1.391121000	-0.880904000
B	1.436147000	-0.868948000	2.316173000
B	-1.382721000	-0.880581000	-2.298734000
B	1.439533000	-0.868353000	-2.315810000
B	0.905315000	-2.280349000	-1.372839000
B	0.012975000	-1.662581000	2.617760000
B	-2.346119000	-1.397510000	0.897847000

B	-0.899678000	-2.290314000	-1.386616000
B	0.904038000	-2.280769000	1.372706000
B	-1.755685000	-2.663064000	-0.002581000
B	-0.901159000	-2.289799000	1.382862000

Electronic energy = -31177.39350617 (Eh)
Gibbs free energy = -31177.28585943 (Eh)
Enthalpy = -31177.21229808 (Eh)

Pu&B_{36}

Pu	-4.359740000	-0.048367000	0.000168000
B	-2.719466000	-0.034636000	1.754704000
B	2.680289000	0.012195000	-1.705327000
B	1.684885000	2.645301000	-0.000168000
B	-0.007864000	1.659712000	2.630378000
B	-1.383662000	0.847806000	-2.288305000
B	-2.718205000	-0.035402000	-1.753017000
B	2.315474000	1.395536000	-0.889201000
B	-2.352881000	1.360202000	0.898812000
B	2.315088000	1.394330000	0.888109000
B	1.416892000	0.876377000	-2.326126000
B	-1.383408000	0.846711000	2.287497000
B	1.417799000	0.875752000	2.325252000
B	2.681947000	0.012451000	1.706040000
B	0.873524000	2.268855000	1.368189000
B	-0.008673000	1.661489000	-2.630998000
B	-2.352956000	1.360796000	-0.898096000
B	-0.928197000	2.271227000	1.394082000
B	0.872661000	2.269225000	-1.367930000
B	-1.776369000	2.630627000	0.000101000
B	-0.928806000	2.272291000	-1.394373000
B	1.728683000	-2.637119000	0.000362000
B	0.020116000	-1.682369000	-2.630352000
B	-1.373529000	-0.896892000	2.291438000
B	2.339078000	-1.376238000	0.888576000
B	-2.328625000	-1.424425000	-0.897858000
B	2.339376000	-1.377401000	-0.888785000
B	1.435171000	-0.873842000	2.326952000
B	-1.370934000	-0.895583000	-2.289960000
B	1.432892000	-0.873719000	-2.326247000
B	0.913062000	-2.277363000	-1.369751000
B	0.020535000	-1.677964000	2.630143000
B	-2.329569000	-1.424482000	0.899380000
B	-0.888323000	-2.310106000	-1.392653000
B	0.912875000	-2.276196000	1.370685000
B	-1.730772000	-2.684321000	0.000630000
B	-0.887902000	-2.308948000	1.393302000

Electronic energy = -32067.10024330 (Eh)
Gibbs free enthalpy = -32066.99528777 (Eh)
Enthalpy = -32066.91998292 (Eh)

U&B$_{38}$

U	0.009990000	4.244650000	0.382780000
B	0.877997000	-2.304958000	-1.411303000
B	-0.874224000	-2.296530000	-1.404542000
B	-1.662934000	-2.735301000	-0.010487000
B	-0.874563000	-2.319806000	1.382387000
B	0.875914000	-2.325232000	1.386144000
B	1.657827000	-2.740687000	-0.010578000
B	1.410798000	0.804312000	2.257089000
B	1.469815000	-0.940437000	2.323829000
B	0.000709000	-1.635519000	2.617374000
B	-1.467119000	-0.937175000	2.323544000
B	-1.390449000	0.804865000	2.244677000
B	0.007618000	1.652991000	2.612958000
B	0.887572000	2.305672000	-1.249307000
B	-0.882515000	2.297444000	-1.246356000
B	-1.907266000	2.786900000	0.035884000
B	-0.928372000	2.222150000	1.358251000
B	0.954037000	2.229314000	1.372542000
B	1.911129000	2.775255000	0.037588000
B	1.372750000	0.851687000	-2.193222000
B	1.474585000	-0.912384000	-2.323706000
B	0.001458000	-1.584347000	-2.623870000
B	-1.469527000	-0.904537000	-2.319970000
B	-1.363794000	0.850055000	-2.186346000
B	0.003093000	1.707687000	-2.527473000
B	2.340299000	1.370393000	-0.832254000
B	2.760537000	-0.019777000	-1.698686000
B	2.274705000	-1.432365000	-0.857173000
B	2.281805000	-1.449153000	0.853103000
B	2.797370000	-0.071873000	1.725256000
B	2.386159000	1.349426000	0.889619000
B	-2.340251000	1.379511000	-0.833683000
B	-2.760368000	-0.011961000	-1.697791000
B	-2.274053000	-1.423399000	-0.855187000
B	-2.281442000	-1.440664000	0.854981000
B	-2.791567000	-0.060404000	1.728426000
B	-2.378595000	1.360973000	0.891940000
B	-2.865650000	-0.038197000	0.014331000
B	2.869437000	-0.049546000	0.012150000

Electronic energy = -30356.83355473 (Eh)
Gibbs free enthalpy = -30356.71460740 (Eh)

Enthalpy = -30356.64113755 (Eh)

$Np\&B_{38}$

Np	-0.004219000	4.306556000	-0.085064000
B	0.882292000	-2.296994000	-1.394413000
B	-0.878113000	-2.297596000	-1.394609000
B	-1.659049000	-2.715784000	0.003881000
B	-0.873586000	-2.290501000	1.402278000
B	0.879417000	-2.291751000	1.405233000
B	1.662504000	-2.713935000	0.005484000
B	1.388581000	0.854756000	2.237280000
B	1.461876000	-0.895866000	2.321024000
B	0.001564000	-1.597869000	2.629501000
B	-1.460059000	-0.897630000	2.319782000
B	-1.388794000	0.852583000	2.235281000
B	-0.000927000	1.693734000	2.585308000
B	0.905430000	2.295279000	-1.333416000
B	-0.910066000	2.294061000	-1.333747000
B	-1.872149000	2.770409000	0.001572000
B	-0.901086000	2.300225000	1.326779000
B	0.898442000	2.300093000	1.325989000
B	1.866606000	2.773981000	-0.000185000
B	1.390996000	0.849418000	-2.239801000
B	1.463164000	-0.899615000	-2.311880000
B	0.002040000	-1.600619000	-2.615487000
B	-1.458877000	-0.899660000	-2.311006000
B	-1.391806000	0.847837000	-2.240137000
B	-0.000636000	1.692483000	-2.592646000
B	2.344484000	1.378002000	-0.858819000
B	2.779431000	-0.015340000	-1.708378000
B	2.279295000	-1.412856000	-0.849697000
B	2.280906000	-1.411288000	0.861681000
B	2.775958000	-0.010334000	1.714756000
B	2.347972000	1.387004000	0.865323000
B	-2.346438000	1.374800000	-0.859393000
B	-2.779213000	-0.018194000	-1.708806000
B	-2.277346000	-1.415197000	-0.850372000
B	-2.278061000	-1.414115000	0.861206000
B	-2.776279000	-0.014681000	1.714381000
B	-2.351404000	1.384100000	0.865808000
B	-2.863496000	-0.022481000	0.002833000
B	2.862264000	-0.018986000	0.002506000

Electronic energy = -31227.02656232 (Eh)

Gibbs free energy = -31226.90774754 (Eh)

Enthalpy = -31226.83486287 (Eh)

$Pu\&B_{38}$

Pu	-0.003750000	4.304843000	-0.187348000
B	0.884271000	-2.287558000	-1.398613000
B	-0.879844000	-2.288010000	-1.397758000
B	-1.660780000	-2.711723000	-0.002054000
B	-0.878233000	-2.290324000	1.401929000
B	0.881070000	-2.288845000	1.401753000
B	1.664736000	-2.709304000	-0.001780000
B	1.379840000	0.846252000	2.238176000
B	1.464853000	-0.905505000	2.324661000
B	0.000718000	-1.601511000	2.628314000
B	-1.463890000	-0.907622000	2.324171000
B	-1.381503000	0.844728000	2.238003000
B	-0.001383000	1.692506000	2.586519000
B	0.917428000	2.265099000	-1.321237000
B	-0.920224000	2.262065000	-1.320972000
B	-1.872386000	2.772147000	0.013924000
B	-0.894254000	2.303299000	1.324788000
B	0.891565000	2.304053000	1.325503000
B	1.868857000	2.775308000	0.015542000
B	1.401176000	0.854389000	-2.227675000
B	1.463503000	-0.884209000	-2.307266000
B	0.001734000	-1.589444000	-2.616117000
B	-1.461485000	-0.886344000	-2.307288000
B	-1.401715000	0.851603000	-2.227811000
B	-0.001062000	1.694009000	-2.581490000
B	2.365533000	1.387964000	-0.856212000
B	2.800561000	-0.015016000	-1.706006000
B	2.284713000	-1.406879000	-0.851948000
B	2.279342000	-1.412827000	0.860214000
B	2.771117000	-0.010774000	1.715069000
B	2.335645000	1.381240000	0.871687000
B	-2.366815000	1.383772000	-0.856832000
B	-2.800552000	-0.019553000	-1.706499000
B	-2.281747000	-1.410070000	-0.852129000
B	-2.277381000	-1.416110000	0.859750000
B	-2.770743000	-0.014513000	1.714349000
B	-2.337377000	1.377940000	0.870926000
B	-2.879039000	-0.022711000	0.005502000
B	2.879121000	-0.018337000	0.006283000

Electronic energy = -32116.73096649 (Eh)

Gibbs free energy = -32116.61020260 (Eh)

Enthalpy = -32116.53746701 (Eh)

$U\&B_{40}$

U	0.000999000	4.293696000	0.053959000

B	-1.904503000	2.880044000	0.372044000
B	-1.681442000	-0.018626000	-2.870937000
B	-1.465828000	2.378526000	-1.196283000
B	1.777930000	-2.796545000	0.394142000
B	0.000413000	-1.710157000	2.890545000
B	-2.643988000	-0.004588000	1.979689000
B	2.388980000	-0.910056000	-1.700415000
B	2.385623000	1.400569000	1.157944000
B	1.906856000	2.882023000	0.374548000
B	1.681967000	-0.017624000	-2.869934000
B	1.461808000	2.379649000	-1.191450000
B	-2.754290000	-1.778719000	-0.389736000
B	-1.399282000	-2.416120000	-1.200239000
B	1.399290000	-2.416644000	-1.200336000
B	0.882232000	-2.395104000	1.687180000
B	-0.852632000	2.364294000	1.598496000
B	2.378206000	0.881243000	-1.681704000
B	-0.881426000	-2.393240000	1.686425000
B	-0.000062000	-2.705739000	-1.994613000
B	-2.830437000	1.748822000	-0.409575000
B	-2.377630000	0.879230000	-1.682117000
B	-2.389861000	-0.910497000	-1.701322000
B	2.377216000	-1.423100000	1.203479000
B	-2.385830000	1.401154000	1.158685000
B	-2.377035000	-1.420714000	1.202821000
B	0.884199000	-1.423014000	-2.540400000
B	-0.884309000	-1.424280000	-2.542049000
B	-1.778137000	-2.794724000	0.394342000
B	-1.384833000	-0.890313000	2.532111000
B	2.643922000	-0.005819000	1.977903000
B	0.852801000	2.362384000	1.597776000
B	0.852921000	1.369689000	-2.476606000
B	-0.853126000	1.369474000	-2.478272000
B	-1.410008000	0.919423000	2.526358000
B	2.753516000	-1.779110000	-0.389100000
B	-0.001129000	2.758073000	-1.939244000
B	1.386946000	-0.892392000	2.533091000
B	0.000544000	1.694511000	2.870290000
B	2.831261000	1.750013000	-0.409112000
B	1.409987000	0.917555000	2.525489000

Electronic energy = -30406.46713687 (Eh)

Gibbs free energy = -30406.34113935 (Eh)

Enthalpy = -30406.26396166 (Eh)

Np&B_{40}

Np	-0.000091000	4.307162000	0.048097000

B	-1.899750000	2.877175000	0.378523000
B	-1.679137000	-0.018500000	-2.867009000
B	-1.458221000	2.381773000	-1.191650000
B	1.778177000	-2.798466000	0.393757000
B	-0.000062000	-1.714488000	2.892958000
B	-2.639150000	-0.004371000	1.974814000
B	2.393040000	-0.913881000	-1.706129000
B	2.382867000	1.404565000	1.162291000
B	1.900383000	2.877430000	0.378472000
B	1.679251000	-0.018561000	-2.866750000
B	1.458718000	2.381882000	-1.191927000
B	-2.749869000	-1.777039000	-0.392121000
B	-1.397474000	-2.422720000	-1.201368000
B	1.397017000	-2.422816000	-1.201195000
B	0.881098000	-2.395740000	1.684933000
B	-0.850949000	2.359589000	1.598755000
B	2.373637000	0.879187000	-1.677868000
B	-0.880994000	-2.395641000	1.684678000
B	-0.000121000	-2.712075000	-1.995899000
B	-2.825621000	1.753123000	-0.408104000
B	-2.373292000	0.879862000	-1.678572000
B	-2.392908000	-0.913670000	-1.706321000
B	2.372539000	-1.422379000	1.200404000
B	-2.382543000	1.404442000	1.162270000
B	-2.372579000	-1.421831000	1.200174000
B	0.882577000	-1.425787000	-2.538020000
B	-0.882543000	-1.425570000	-2.538021000
B	-1.778344000	-2.798235000	0.393686000
B	-1.378697000	-0.888147000	2.529836000
B	2.639280000	-0.004568000	1.974223000
B	0.851749000	2.360232000	1.599360000
B	0.853226000	1.372127000	-2.479500000
B	-0.852711000	1.372028000	-2.479458000
B	-1.411426000	0.920760000	2.532658000
B	2.749749000	-1.777362000	-0.392052000
B	0.000237000	2.756056000	-1.943984000
B	1.378509000	-0.887869000	2.529789000
B	0.000179000	1.699159000	2.874864000
B	2.825934000	1.753152000	-0.408000000
B	1.411758000	0.920641000	2.532448000

Electronic energy = -31276.67335107 (Eh)
Gibbs free energy = -31276.54785068 (Eh)
Enthalpy = -31276.47053429 (Eh)

Pu&B_{40}

Pu	0.005638000	4.337818000	0.043840000

B	-1.875282000	2.868211000	0.404433000
B	-1.677524000	0.012693000	-2.868256000
B	-1.442157000	2.413517000	-1.180897000
B	1.774836000	-2.780845000	0.386826000
B	0.000582000	-1.714215000	2.898194000
B	-2.634913000	0.004089000	1.983941000
B	2.393671000	-0.890750000	-1.703836000
B	2.376473000	1.412914000	1.187467000
B	1.882708000	2.864628000	0.404520000
B	1.680207000	0.009714000	-2.867549000
B	1.449313000	2.410612000	-1.180787000
B	-2.749845000	-1.751619000	-0.391133000
B	-1.403446000	-2.390566000	-1.209378000
B	1.403208000	-2.393896000	-1.209564000
B	0.881638000	-2.386132000	1.682082000
B	-0.850876000	2.373516000	1.642129000
B	2.368808000	0.897106000	-1.672044000
B	-0.882320000	-2.385549000	1.682895000
B	-0.000373000	-2.680617000	-1.998215000
B	-2.802890000	1.763476000	-0.388610000
B	-2.364118000	0.900271000	-1.672392000
B	-2.390738000	-0.887380000	-1.703096000
B	2.374284000	-1.416169000	1.206295000
B	-2.370532000	1.418341000	1.189094000
B	-2.372997000	-1.412828000	1.206885000
B	0.884199000	-1.399652000	-2.545396000
B	-0.883324000	-1.398970000	-2.547163000
B	-1.775438000	-2.778454000	0.386901000
B	-1.378503000	-0.888419000	2.540905000
B	2.638796000	0.000012000	1.984380000
B	0.858512000	2.372494000	1.642160000
B	0.855647000	1.399261000	-2.473195000
B	-0.849673000	1.400423000	-2.473489000
B	-1.406066000	0.917698000	2.558068000
B	2.749865000	-1.755312000	-0.391581000
B	0.003721000	2.795708000	-1.950135000
B	1.379697000	-0.889784000	2.540147000
B	0.003304000	1.703086000	2.907499000
B	2.809360000	1.759839000	-0.389213000
B	1.411394000	0.915757000	2.557309000

Electronic energy = -32166.37777647 (Eh)

Gibbs free enthalpy = -32166.25558594 (Eh)

Enthalpy = -32166.17612083 (Eh)

U@B_{36}

U	0.001571000	-0.004640000	0.001051000

B	-2.662837000	-0.014909000	1.620396000
B	2.669155000	0.004466000	-1.617574000
B	1.593537000	2.623677000	-0.000776000
B	0.000134000	1.592082000	2.669735000
B	-1.440910000	0.865716000	-2.330142000
B	-2.668576000	-0.015634000	-1.607491000
B	2.332454000	1.429254000	-0.890958000
B	-2.338407000	1.413253000	0.894697000
B	2.335779000	1.430869000	0.887890000
B	1.431905000	0.876704000	-2.335311000
B	-1.433478000	0.866568000	2.339866000
B	1.438629000	0.877494000	2.334226000
B	2.672657000	0.005056000	1.612108000
B	0.879156000	2.296152000	1.437765000
B	-0.008067000	1.591655000	-2.667586000
B	-2.342440000	1.413300000	-0.883875000
B	-0.888339000	2.290746000	1.440903000
B	0.874604000	2.294575000	-1.436925000
B	-1.608764000	2.612531000	0.003891000
B	-0.892374000	2.288565000	-1.435039000
B	1.613795000	-2.622116000	-0.000293000
B	0.003768000	-1.602301000	-2.666232000
B	-1.426397000	-0.887304000	2.337843000
B	2.345972000	-1.422820000	0.888087000
B	-2.330777000	-1.441684000	-0.883005000
B	2.343747000	-1.423425000	-0.891370000
B	1.445212000	-0.876523000	2.334920000
B	-1.434736000	-0.887862000	-2.331127000
B	1.437943000	-0.876747000	-2.334982000
B	0.891764000	-2.298633000	-1.436156000
B	0.012317000	-1.603070000	2.671401000
B	-2.327909000	-1.441053000	0.894921000
B	-0.874683000	-2.306352000	-1.433296000
B	0.896958000	-2.299733000	1.438589000
B	-1.588929000	-2.635615000	0.004931000
B	-0.870170000	-2.307381000	1.441786000

Electronic energy = -30307.44269320 (Eh)

Gibbs free energy = -30307.32501203 (Eh)

Enthalpy = -30307.25675192 (Eh)

Np@B_{36}

Np	0.005275000	-0.002106000	-0.001311000
B	-2.648714000	-0.002744000	1.598936000
B	2.659336000	0.000856000	-1.600709000
B	1.605484000	2.652976000	0.000373000
B	0.001570000	1.602408000	2.652323000

B	-1.424737000	0.879537000	-2.323323000
B	-2.646212000	-0.002714000	-1.604695000
B	2.326537000	1.431804000	-0.880142000
B	-2.318592000	1.429058000	0.878181000
B	2.325360000	1.432427000	0.882203000
B	1.437653000	0.881373000	-2.321355000
B	-1.428474000	0.879442000	2.319444000
B	1.433561000	0.881317000	2.321545000
B	2.656581000	0.000967000	1.602544000
B	0.883335000	2.321326000	1.430890000
B	0.006037000	1.602164000	-2.654475000
B	-2.317844000	1.429387000	-0.883816000
B	-0.878710000	2.320778000	1.430196000
B	0.885692000	2.321422000	-1.431467000
B	-1.598390000	2.650701000	-0.002257000
B	-0.876503000	2.320347000	-1.433580000
B	1.608788000	-2.651773000	0.000132000
B	0.007999000	-1.601528000	-2.652437000
B	-1.428278000	-0.883332000	2.320924000
B	2.327964000	-1.430943000	0.882173000
B	-2.315934000	-1.434507000	-0.884201000
B	2.329514000	-1.431132000	-0.880805000
B	1.436272000	-0.881179000	2.322551000
B	-1.424975000	-0.883345000	-2.325407000
B	1.439874000	-0.881344000	-2.322128000
B	0.889025000	-2.322363000	-1.431973000
B	0.003888000	-1.601849000	2.650607000
B	-2.317588000	-1.434195000	0.878178000
B	-0.873525000	-2.323679000	-1.433331000
B	0.886625000	-2.322481000	1.431060000
B	-1.594998000	-2.654238000	-0.002405000
B	-0.875888000	-2.323419000	1.429831000

Electronic energy = -31177.60869626 (Eh)
Gibbs free energy = -31177.49247503 (Eh)
Enthalpy = -31177.42388500 (Eh)

Pu@B_{36}

Pu	-0.003159000	-0.007175000	0.009841000
B	-2.651729000	-0.001095000	1.606401000
B	2.645657000	-0.013260000	-1.586982000
B	1.592631000	2.642068000	0.015252000
B	0.001657000	1.612593000	2.670514000
B	-1.458237000	0.888188000	-2.328182000
B	-2.664408000	-0.003366000	-1.608973000
B	2.315430000	1.423118000	-0.871841000
B	-2.341252000	1.447128000	0.905949000

B	2.311720000	1.419745000	0.900703000
B	1.426442000	0.873625000	-2.309608000
B	-1.429155000	0.884536000	2.324775000
B	1.426642000	0.875912000	2.333482000
B	2.658222000	-0.010993000	1.628589000
B	0.892789000	2.331309000	1.463733000
B	-0.010112000	1.588545000	-2.639491000
B	-2.327627000	1.421758000	-0.872471000
B	-0.885679000	2.317051000	1.439006000
B	0.877190000	2.311653000	-1.420899000
B	-1.622818000	2.653589000	0.014159000
B	-0.895378000	2.307339000	-1.416849000
B	1.616665000	-2.667943000	0.005383000
B	-0.007882000	-1.626905000	-2.650971000
B	-1.432611000	-0.887965000	2.329116000
B	2.321493000	-1.436153000	0.892077000
B	-2.317947000	-1.434121000	-0.881134000
B	2.335140000	-1.461509000	-0.886471000
B	1.452030000	-0.902609000	2.347733000
B	-1.432914000	-0.890307000	-2.313932000
B	1.423033000	-0.898987000	-2.305255000
B	0.879497000	-2.331438000	-1.419479000
B	0.003887000	-1.602904000	2.658989000
B	-2.321634000	-1.437516000	0.891391000
B	-0.899005000	-2.345679000	-1.444165000
B	0.889190000	-2.321684000	1.436385000
B	-1.598893000	-2.656509000	0.004309000
B	-0.883378000	-2.326041000	1.440412000

Electronic energy = -32067.28932244 (Eh)
Gibbs free energy = -32067.17670549 (Eh)
Enthalpy = -32067.10656526 (Eh)

U@B_{38}

U	0.023664000	-0.032003000	0.009667000
B	0.858315000	-2.316500000	-1.410812000
B	-0.876756000	-2.313915000	-1.411349000
B	-1.654804000	-2.738706000	-0.021922000
B	-0.875068000	-2.345224000	1.375403000
B	0.859730000	-2.350008000	1.375150000
B	1.635872000	-2.745842000	-0.023034000
B	1.386808000	0.790948000	2.303555000
B	1.382293000	-0.911377000	2.282250000
B	-0.007029000	-1.710170000	2.653376000
B	-1.392440000	-0.905040000	2.277340000
B	-1.390571000	0.792906000	2.297226000
B	-0.001657000	1.582688000	2.692265000

B	0.865257000	2.283786000	-1.356328000
B	-0.869543000	2.286600000	-1.356509000
B	-1.645855000	2.680249000	0.042880000
B	-0.868116000	2.253192000	1.431274000
B	0.866600000	2.250458000	1.429746000
B	1.643791000	2.677875000	0.041522000
B	1.384570000	0.845002000	-2.265068000
B	1.381245000	-0.857076000	-2.284976000
B	-0.009300000	-1.647605000	-2.673343000
B	-1.394595000	-0.850669000	-2.277023000
B	-1.393281000	0.846742000	-2.257203000
B	-0.004597000	1.647299000	-2.634029000
B	2.285496000	1.384017000	-0.852846000
B	2.804177000	-0.015594000	-1.695884000
B	2.281277000	-1.432470000	-0.885839000
B	2.282767000	-1.453489000	0.871277000
B	2.804987000	-0.055860000	1.713004000
B	2.287455000	1.362829000	0.904015000
B	-2.292007000	1.386232000	-0.850001000
B	-2.829138000	-0.006931000	-1.697606000
B	-2.297094000	-1.421848000	-0.883956000
B	-2.295714000	-1.442360000	0.871454000
B	-2.825919000	-0.047745000	1.718399000
B	-2.291565000	1.365478000	0.904896000
B	-2.787078000	-0.027439000	0.011144000
B	2.779548000	-0.035647000	0.008820000

Electronic energy = -30357.01264628 (Eh)
Gibbs free energy = -30356.88877217 (Eh)
Enthalpy = -30356.81998006 (Eh)

Np@B_{38}

Np	0.001035000	-0.006138000	0.001595000
B	0.863501000	-2.313304000	-1.385288000
B	-0.859636000	-2.314309000	-1.385218000
B	-1.648418000	-2.729929000	0.005688000
B	-0.859743000	-2.310569000	1.395692000
B	0.863103000	-2.309429000	1.395826000
B	1.652321000	-2.727574000	0.005926000
B	1.399811000	0.862937000	2.296172000
B	1.402162000	-0.860724000	2.299999000
B	0.001186000	-1.640972000	2.668947000
B	-1.400872000	-0.862688000	2.299736000
B	-1.400722000	0.860745000	2.296066000
B	-0.000932000	1.644141000	2.663950000
B	0.860302000	2.306444000	-1.391679000
B	-0.863739000	2.304875000	-1.392189000

B	-1.652095000	2.723143000	-0.001515000
B	-0.863970000	2.308540000	1.390324000
B	0.860338000	2.311292000	1.390234000
B	1.648865000	2.725567000	-0.001290000
B	1.400085000	0.856826000	-2.294360000
B	1.402620000	-0.867089000	-2.293284000
B	0.001497000	-1.648065000	-2.660028000
B	-1.400664000	-0.868943000	-2.293277000
B	-1.400523000	0.854663000	-2.294316000
B	-0.000880000	1.637296000	-2.664501000
B	2.276323000	1.411061000	-0.871208000
B	2.792751000	-0.002938000	-1.704675000
B	2.279834000	-1.415910000	-0.868231000
B	2.279780000	-1.413553000	0.876814000
B	2.793275000	0.001719000	1.709784000
B	2.276227000	1.413145000	0.872357000
B	-2.278191000	1.407826000	-0.871506000
B	-2.792743000	-0.007028000	-1.704939000
B	-2.277357000	-1.419041000	-0.868422000
B	-2.277425000	-1.416794000	0.876613000
B	-2.793227000	-0.002387000	1.709080000
B	-2.278419000	1.410101000	0.871861000
B	-2.786988000	-0.004718000	0.001991000
B	2.787362000	-0.000746000	0.002364000

Electronic energy = -31227.16716504 (Eh)
Gibbs free energy = -31227.04423717 (Eh)
Enthalpy = -31226.97411127 (Eh)

Pu@B_{38}

Pu	0.003071000	-0.027287000	0.003895000
B	0.871213000	-2.350248000	-1.392860000
B	-0.854683000	-2.356945000	-1.385411000
B	-1.655327000	-2.768140000	0.004875000
B	-0.861197000	-2.352123000	1.396499000
B	0.865094000	-2.359001000	1.392769000
B	1.662936000	-2.765507000	-0.000076000
B	1.389808000	0.823621000	2.305861000
B	1.388685000	-0.906811000	2.301599000
B	0.002718000	-1.712259000	2.676048000
B	-1.382159000	-0.900316000	2.294351000
B	-1.390093000	0.817445000	2.300597000
B	-0.000309000	1.621644000	2.679355000
B	0.867528000	2.272941000	-1.389420000
B	-0.858975000	2.276428000	-1.384282000
B	-1.657231000	2.684363000	0.008238000
B	-0.865151000	2.268652000	1.401372000

B	0.860231000	2.276346000	1.397725000
B	1.657480000	2.687092000	0.005442000
B	1.395000000	0.823695000	-2.289870000
B	1.389325000	-0.894620000	-2.287345000
B	0.005783000	-1.709043000	-2.669757000
B	-1.379493000	-0.903706000	-2.293482000
B	-1.386366000	0.825491000	-2.295234000
B	0.003183000	1.624815000	-2.666837000
B	2.289950000	1.380873000	-0.876013000
B	2.804175000	-0.037170000	-1.713014000
B	2.290313000	-1.455188000	-0.877851000
B	2.280140000	-1.458245000	0.873604000
B	2.758173000	-0.039056000	1.695734000
B	2.279393000	1.380524000	0.876506000
B	-2.276793000	1.378335000	-0.865013000
B	-2.752327000	-0.041884000	-1.685759000
B	-2.272295000	-1.460670000	-0.866897000
B	-2.283061000	-1.460464000	0.885657000
B	-2.798950000	-0.044037000	1.722924000
B	-2.286177000	1.376062000	0.887738000
B	-2.776099000	-0.041741000	0.010644000
B	2.781856000	-0.037182000	-0.001435000

Electronic energy = -32116.84680577 (Eh)
Gibbs free energy = -32116.72211989 (Eh)
Enthalpy = -32116.65225477 (Eh)

U@B_{40}

U	0.001595000	-0.004749000	-0.003708000
B	-1.705205000	2.747833000	0.363730000
B	-1.641875000	-0.003061000	-2.897884000
B	-1.450671000	2.409233000	-1.239820000
B	1.701630000	-2.739963000	0.374177000
B	0.000267000	-1.633072000	2.896224000
B	-2.662501000	0.006667000	1.906343000
B	2.365266000	-0.868490000	-1.677871000
B	2.403342000	1.456793000	1.224225000
B	1.704920000	2.748223000	0.362474000
B	1.641692000	-0.003603000	-2.897851000
B	1.452537000	2.408352000	-1.240652000
B	-2.743382000	-1.699485000	-0.370466000
B	-1.451633000	-2.406111000	-1.229593000
B	1.452306000	-2.406815000	-1.230481000
B	0.867045000	-2.359421000	1.678657000
B	-0.867005000	2.380816000	1.669970000
B	2.365914000	0.866332000	-1.681745000
B	-0.867620000	-2.359683000	1.679560000

B	0.000015000	-2.663655000	-1.906567000
B	-2.744408000	1.707198000	-0.379890000
B	-2.365742000	0.867554000	-1.681926000
B	-2.364408000	-0.867610000	-1.676994000
B	2.406210000	-1.446497000	1.231957000
B	-2.400858000	1.453844000	1.222713000
B	-2.406795000	-1.447419000	1.233549000
B	0.870003000	-1.399596000	-2.549142000
B	-0.869854000	-1.399281000	-2.548586000
B	-1.702147000	-2.739981000	0.375032000
B	-1.395691000	-0.861486000	2.546880000
B	2.661377000	0.007318000	1.904696000
B	0.865871000	2.373087000	1.666397000
B	0.870469000	1.393769000	-2.553696000
B	-0.870135000	1.394246000	-2.553841000
B	-1.397387000	0.879105000	2.542771000
B	2.744223000	-1.700427000	-0.371321000
B	0.000622000	2.661106000	-1.918928000
B	1.396318000	-0.861738000	2.545871000
B	-0.001473000	1.649239000	2.885892000
B	2.744316000	1.705085000	-0.379309000
B	1.396676000	0.878628000	2.543706000

Electronic energy = -30406.61836681 (Eh)
Gibbs free energy = -30406.49345010 (Eh)
Enthalpy = -30406.41439024 (Eh)

$Np@B_{40}$

Np	0.072910000	0.000820000	-0.380488000
B	-1.683347000	2.715364000	0.387682000
B	-1.628128000	-0.013248000	-2.947490000
B	-1.414825000	2.394112000	-1.251002000
B	1.668231000	-2.723491000	0.381606000
B	-0.005412000	-1.698173000	2.999667000
B	-2.633640000	0.001711000	2.022877000
B	2.348049000	-0.891959000	-1.733851000
B	2.340742000	1.437653000	1.250566000
B	1.671958000	2.719333000	0.373844000
B	1.642586000	-0.012922000	-2.964452000
B	1.434651000	2.398936000	-1.269665000
B	-2.675583000	-1.680490000	-0.364018000
B	-1.416053000	-2.406661000	-1.236757000
B	1.437452000	-2.411962000	-1.262055000
B	0.845224000	-2.351464000	1.712130000
B	-0.870606000	2.359379000	1.729504000
B	2.341952000	0.866002000	-1.730479000
B	-0.872714000	-2.359559000	1.745375000

B	0.004213000	-2.704091000	-1.969673000
B	-2.669572000	1.673027000	-0.369825000
B	-2.306693000	0.862862000	-1.706496000
B	-2.319976000	-0.881734000	-1.708805000
B	2.338940000	-1.437660000	1.250660000
B	-2.354038000	1.421044000	1.256039000
B	-2.356483000	-1.419116000	1.263510000
B	0.874941000	-1.426247000	-2.627198000
B	-0.863109000	-1.429070000	-2.617590000
B	-1.680981000	-2.715430000	0.398201000
B	-1.393057000	-0.884493000	2.653251000
B	2.599220000	-0.000918000	1.975078000
B	0.848114000	2.354898000	1.705368000
B	0.874134000	1.400648000	-2.635333000
B	-0.861557000	1.404459000	-2.626649000
B	-1.391880000	0.887494000	2.652573000
B	2.671615000	-1.678322000	-0.375899000
B	0.005583000	2.686229000	-1.988261000
B	1.373833000	-0.886317000	2.645041000
B	-0.008435000	1.703477000	2.991893000
B	2.664799000	1.662784000	-0.380675000
B	1.361812000	0.879427000	2.626883000

Electronic energy = -31276.77619800 (Eh)

Gibbs free energy = -31276.65242821 (Eh)

Enthalpy = -31276.57344315 (Eh)

Pu@B_{40}

Pu	-0.033497000	-0.050066000	-0.324235000
B	-1.712934000	2.679937000	0.377134000
B	-1.671234000	-0.064348000	-2.940305000
B	-1.453124000	2.350322000	-1.257700000
B	1.659066000	-2.778055000	0.399248000
B	-0.024162000	-1.740382000	2.994547000
B	-2.645860000	-0.041111000	2.002002000
B	2.302055000	-0.936310000	-1.711545000
B	2.325254000	1.385339000	1.242873000
B	1.658053000	2.680890000	0.373312000
B	1.606065000	-0.063986000	-2.944043000
B	1.390628000	2.348573000	-1.259490000
B	-2.708071000	-1.736219000	-0.359761000
B	-1.452484000	-2.461869000	-1.234295000
B	1.390705000	-2.460208000	-1.236584000
B	0.834233000	-2.409065000	1.732346000
B	-0.886199000	2.324599000	1.712557000
B	2.301891000	0.819820000	-1.719920000
B	-0.886583000	-2.409348000	1.735434000

B	-0.031303000	-2.762320000	-1.970641000
B	-2.708367000	1.632073000	-0.376258000
B	-2.365450000	0.820361000	-1.715742000
B	-2.364681000	-0.936852000	-1.706435000
B	2.325833000	-1.473925000	1.256320000
B	-2.378701000	1.384830000	1.248720000
B	-2.378807000	-1.474200000	1.262589000
B	0.838391000	-1.480748000	-2.608200000
B	-0.903324000	-1.481061000	-2.605701000
B	-1.712736000	-2.776968000	0.402962000
B	-1.405159000	-0.923088000	2.639907000
B	2.594628000	-0.040834000	1.994745000
B	0.834523000	2.324764000	1.710613000
B	0.837977000	1.355680000	-2.621641000
B	-0.903231000	1.355701000	-2.619488000
B	-1.405150000	0.846931000	2.631338000
B	2.651154000	-1.737164000	-0.367027000
B	-0.031522000	2.643706000	-1.996248000
B	1.355729000	-0.922932000	2.635901000
B	-0.024285000	1.667533000	2.978637000
B	2.650185000	1.632692000	-0.382686000
B	1.355917000	0.847095000	2.627614000

Electronic energy = -32166.48572106 (Eh)

Gibbs free energy = -32166.36347378 (Eh)

Enthalpy = -32166.28355673 (Eh)

Table S2. Selected bond lengths [Å], fuzzy bond order (FBO) and VDD charge (*e*) of An&B_m (An = U, Np, Pu; *m* = 28, 32, 34, 36, 38, 40).

Species	2S+1	bond	*r*(Å)	FBO	*Q*(An)(*e*)	*Q*(B)(*e*)
UB_{28}	3	U-B_8	2.500	0.944	0.635	-0.151
		U-B_{18}	2.482	0.868		-0.096
		U-B_{15}	2.512	0.880		-0.108
UB_{32}	3	U-B_4	2.467	0.978	0.664	-0.124
		U-B_{19}	2.428	1.053		-0.123
		U-B_{12}	2.436	0.954		-0.235
UB_{34}	3	U-B_6	2.497	0.876	0.671	-0.098
		U-B_{18}	2.540	0.893		-0.141
		U-B_{24}	2.453	0.866		-0.057
		U-B_{16}	2.504	0.862		-0.101
UB_{36}	3	U-B_1	2.359	1.038	0.700	-0.225
		U-B_6	2.356	1.057		-0.224
UB_{38}		U-B_{15}	2.433	1.022	0.673	-0.205

		U-B_{16}	2.432	0.845		-0.053
		U-B_{17}	2.434	0.850		-0.057
		U-B_{18}	2.427	1.013		-0.198
UB_{40}	3	U-B_{1}	2.394	1.010	0.639	-0.189
		U-B_{9}	2.393	1.013		-0.190
		U-B_{36}	2.516	0.935		-0.193
NpB_{28}	4	Np-B_{8}	2.521	0.875	0.596	-0.133
		Np-B_{18}	2.493	0.828		-0.079
		Np-B_{15}	2.523	0.835		3.905
NpB_{32}	4	Np-B_{4}	2.481	0.962	0.615	-0.139
		Np-B_{19}	2.453	0.955		-0.095
		Np-B12	2.444	0.908		-0.220
NpB_{34}	4	Np-B_{6}	2.505	0.845	0.617	-0.093
		Np-B_{18}	2.558	0.839		-0.127
		Np-B_{24}	2.477	0.819		-0.036
		Np-B_{16}	2.522	0.830		-0.102
NpB_{36}	4	Np-B_{1}	2.393	0.970	0.634	-0.210
		Np-B_{6}	2.393	0.970		-0.210
NpB_{38}	4	Np-B_{15}	2.420	0.962	0.625	-0.191
		Np-B_{18}	2.420	0.963		-0.191
NpB_{40}	4	Np-B_{1}	2.401	0.973	0.589	-0.179
		Np-B_{9}	2.401	0.973		-0.179
		Np-B_{36}	2.525	0.905		-0.188
PuB_{28}	5	Pu-B_{8}	2.553	0.838	0.726	-0.144
		Pu-B_{18}	2.500	0.805		-0.089
		Pu-B_{15}	2.536	0.805		-0.107
PuB_{32}	5	Pu-B_{4}	2.545	0.863	0.732	-0.109
		Pu-B_{19}	2.475	0.921		-0.128
		Pu-B_{12}	2.437	0.913		-0.241
PuB_{34}	5	Pu-B_{6}	2.502	0.835	0.733	-0.109
		Pu-B_{4}	2.546	0.728		-0.056
		Pu-B_{18}	2.582	0.803		-0.144
		Pu-B_{24}	2.503	0.780		-0.028
		Pu-B_{16}	2.520	0.830		-0.114
PuB_{36}	5	Pu-B_{1}	2.402	0.966	0.740	-0.229
		Pu-B_{6}	2.402	0.965		-0.228
PuB_{38}	5	Pu-B_{15}	2.425	0.954	0.747	-0.218

		Pu-B_{18}	2.425	0.955		-0.218
PuB_{40}	5	Pu-B_1	2.414	0.925	0.696	-0.192
		Pu-B_9	2.413	0.925		-0.192
		Pu-B_{36}	2.521	0.914		-0.212

Table S3. Topological parameters for the An–B bond critical points (BCPs) of the An&B_m (An = U, Np, Pu; m = 28, 32, 34, 36, 38, 40) at PBE-ZORA/def2-TZVPP level.

Species	Bond	$\rho(r)$	$\nabla^2\rho(r)$	$G(r)$	$V(r)$	$H(r)$	$-V(r)/G(r)$	ELF
UB_{28}	UB_8	0.070	0.081	0.040	-0.060	-0.020	1.498	0.421
	UB_{18}	0.071	0.076	0.040	-0.060	-0.021	1.523	0.438
	UB_{15}	0.070	0.076	0.039	-0.059	-0.020	1.516	0.437
UB_{32}	UB_4	0.073	0.088	0.044	-0.066	-0.022	1.500	0.404
	UB_{19}	0.077	0.082	0.045	-0.070	-0.025	1.546	0.447
	UB_{12}	0.077	0.056	0.040	-0.065	-0.026	1.649	0.502
UB_{34}	UB_6	0.068	0.083	0.040	-0.060	-0.019	1.483	0.396
	UB_{18}	0.064	0.096	0.040	-0.057	-0.016	1.407	0.347
	UB_{24}	0.077	0.050	0.038	-0.064	-0.026	1.675	0.519
	UB_{16}	0.068	0.086	0.040	-0.059	-0.019	1.465	0.391
UB_{36}	U-B_1	0.084	0.090	0.052	-0.082	-0.030	1.569	0.446
	U-B_6	0.084	0.092	0.053	-0.082	-0.029	1.560	0.440
UB_{38}	UB_{15}	0.073	0.096	0.047	-0.069	-0.023	1.487	0.378
	UB_{16}	0.079	0.065	0.042	-0.068	-0.026	1.615	0.494
	UB_{17}	0.076	0.080	0.044	-0.069	-0.024	1.549	0.440
	UB_{18}	0.074	0.090	0.046	-0.069	-0.023	1.509	0.398
UB_{40}	UB_1	0.081	0.059	0.044	-0.072	-0.029	1.662	0.505
	UB_9	0.081	0.062	0.044	-0.073	-0.029	1.649	0.496
	UB_{36}	0.067	0.063	0.035	-0.055	-0.020	1.555	0.449
NpB_{28}	NpB_8	0.065	0.091	0.040	-0.057	-0.017	1.425	0.364
	NpB_{18}	0.066	0.089	0.040	-0.058	-0.018	1.447	0.376
	NpB_{15}	0.066	0.085	0.039	-0.057	-0.018	1.454	0.390
NpB_{32}	NpB_4	0.070	0.092	0.043	-0.063	-0.020	1.463	0.385
	NpB_{19}	0.071	0.092	0.044	-0.065	-0.021	1.475	0.395
	NpB_{12}	0.073	0.066	0.040	-0.063	-0.023	1.583	0.460
NpB_{34}	NpB_6	0.065	0.087	0.039	-0.057	-0.018	1.451	0.377
	NpB_{18}	0.060	0.102	0.040	-0.054	-0.014	1.358	0.306
	NpB_{24}	0.071	0.058	0.037	-0.059	-0.022	1.604	0.476
	NpB_{16}	0.064	0.087	0.038	-0.055	-0.017	1.433	0.373

NpB_{36}	Np-B_1	0.077	0.096	0.049	-0.073	-0.025	1.505	0.406
	Np-B6	0.077	0.096	0.049	-0.073	-0.025	1.505	0.405
NpB_{38}	Np-B_{15}	0.073	0.097	0.047	-0.069	-0.022	1.479	0.385
	Np-B_{18}	0.073	0.098	0.047	-0.069	-0.022	1.476	0.382
NpB_{40}	Np-B_1	0.078	0.066	0.043	-0.069	-0.026	1.616	0.481
	Np-B_9	0.078	0.066	0.044	-0.069	-0.026	1.592	0.481
	Np-B_{36}	0.064	0.070	0.035	-0.053	-0.018	1.503	0.412
PuB_{28}	PuB_8	0.059	0.100	0.039	-0.053	-0.039	1.357	0.311
	PuB_{18}	0.063	0.099	0.041	-0.056	-0.016	1.390	0.333
	PuB_{15}	0.063	0.095	0.039	-0.055	-0.016	1.397	0.346
PuB_{32}	PuB_4	0.061	0.093	0.039	-0.054	-0.015	1.396	0.336
	PuB_{19}	0.067	0.097	0.042	-0.061	-0.018	1.427	0.362
	PuB_{12}	0.072	0.068	0.039	-0.062	-0.022	1.567	0.458
PuB_{34}	PuB_6	0.065	0.085	0.038	-0.056	-0.017	1.449	0.382
	PuB_4	0.058	0.096	0.037	-0.050	-0.013	1.351	0.309
	PuB_{24}	0.065	0.066	0.035	-0.054	-0.019	1.531	0.430
	PuB_{16}	0.061	0.094	0.039	-0.054	-0.015	1.391	0.337
PuB_{36}	Pu-B_1	0.073	0.103	0.048	-0.070	-0.022	1.461	0.375
	Pu-B_6	0.073	0.103	0.048	-0.070	-0.022	1.461	0.375
PuB_{38}	Pu-B_{15}	0.070	0.102	0.046	-0.067	-0.021	1.447	0.361
	Pu-B_{18}	0.070	0.102	0.046	-0.067	-0.021	1.447	0.361
PuB_{40}	Pu-B_1	0.074	0.070	0.041	-0.065	-0.024	1.578	0.458
	Pu-B_9	0.074	0.069	0.041	-0.065	-0.024	1.580	0.460
	Pu-B_{36}	0.061	0.076	0.035	-0.052	-0.016	1.463	0.380

Table S4. Hirshfeld atomic charge of An&B_{36} and An@B_{36} (Heavy elements in bold)

NpB_{36}		PuB_{36}		UB_{36}	
Endohedral	Exohedral	Endohedral	Exohedral	Endohedral	Exohedral
**0.005275**	**-4.343141**	**-0.003166**	**-4.35974**	**0.001571**	**-4.287395**
-2.648714	-2.71829	-2.651731	-2.719466	-2.662837	-2.694428
2.659336	2.695563	2.645637	2.680289	2.669155	2.685843
1.605484	1.731149	1.592585	1.684885	1.593537	1.685645
0.00157	0.009694	0.001651	-0.007864	0.000134	0.009196
-1.424737	-1.356578	-1.458228	-1.383662	-1.44091	-1.369607
-2.646212	-2.716016	-2.664401	-2.718205	-2.668576	-2.715236
2.326537	2.358561	2.315397	2.315474	2.332454	2.32208
-2.318592	-2.361105	-2.341259	-2.352881	-2.338407	-2.336388
2.32536	2.357346	2.311726	2.315088	2.335779	2.312835
1.437653	1.43267	1.426384	1.416892	1.431905	1.421938
-1.428474	-1.359985	-1.429151	-1.383408	-1.433478	-1.37127
1.433561	1.429307	1.426657	1.417799	1.438629	1.425072
2.656581	2.693313	2.65827	2.681947	2.672657	2.678839
0.883335	0.908195	0.892762	0.873524	0.879156	0.887824
0.006037	0.013553	-0.010134	-0.008673	-0.008067	0.001355
-2.317844	-2.359984	-2.327617	-2.352956	-2.34244	-2.342471
-0.87871	-0.903904	-0.885663	-0.928197	-0.888339	-0.917323
0.885692	0.910102	0.877127	0.872661	0.874604	0.876508
-1.59839	-1.769665	-1.622825	-1.776369	-1.608764	-1.737323
-0.876503	-0.90217	-0.895364	-0.928806	-0.892374	-0.923384
1.608788	1.709297	1.616687	1.728683	1.613795	1.713218
0.007999	0.016882	-0.007888	0.020116	0.003768	0.012836
-1.428278	-1.385892	-1.432602	-1.373529	-1.426397	-1.330811
2.327964	2.343479	2.32153	2.339078	2.345972	2.337027
-2.315934	-2.345895	-2.317939	-2.328625	-2.330777	-2.343752
2.329514	2.344737	2.335157	2.339376	2.343747	2.335019
1.436272	1.436147	1.452067	1.435171	1.445212	1.417626
-1.424975	-1.382721	-1.432897	-1.370934	-1.434736	-1.376292
1.439874	1.439533	1.423000	1.432892	1.437943	1.432473
0.889025	0.905315	0.879465	0.913062	0.891764	0.903288
0.003888	0.012975	0.003897	0.020535	0.012317	0.02078
-2.317588	-2.346119	-2.321631	-2.329569	-2.327909	-2.325704
-0.873525	-0.899678	-0.898978	-0.888323	-0.874683	-0.903816
0.886625	0.904038	0.889198	0.912875	0.896958	0.906394
-1.594998	-1.755685	-1.59887	-1.730772	-1.588929	-1.720029
-0.875888	-0.901159	-0.883355	-0.887902	-0.87017	-0.894699

Table S5. Hirshfeld atomic charge of An&B_{38} and An@B_{38} (Heavy elements in bold)

NpB_{38}		PuB_{38}		UB_{38}	
Endohedral	Exohedral	Endohedral	Exohedral	Endohedral	Exohedral
**0.001035**	**-0.004219**	**0.003071**	**-0.00375**	**0.023664**	**0.00999**
0.863501	0.882292	0.871213	0.884271	0.858315	0.877997
-0.859636	-0.878113	-0.854683	-0.879844	-0.876756	-0.874224
-1.648418	-1.659049	-1.655327	-1.66078	-1.654804	-1.662934
-0.859743	-0.873586	-0.861197	-0.878233	-0.875068	-0.874563
0.863103	0.879417	0.865094	0.88107	0.85973	0.875914
1.652321	1.662504	1.662936	1.664736	1.635872	1.657827
1.399811	1.388581	1.389808	1.37984	1.386808	1.410798
1.402162	1.461876	1.388685	1.464853	1.382293	1.469815
0.001186	0.001564	0.002718	0.000718	-0.007029	0.000709
-1.400872	-1.460059	-1.382159	-1.46389	-1.39244	-1.467119
-1.400722	-1.388794	-1.390093	-1.381503	-1.390571	-1.390449
-0.000932	-0.000927	-0.000309	-0.001383	-0.001657	0.007618
0.860302	0.90543	0.867528	0.917428	0.865257	0.887572
-0.863739	-0.910066	-0.858975	-0.920224	-0.869543	-0.882515
-1.652095	-1.872149	-1.657231	-1.872386	-1.645855	-1.907266
-0.86397	-0.901086	-0.865151	-0.894254	-0.868116	-0.928372
0.860338	0.898442	0.860231	0.891565	0.8666	0.954037
1.648865	1.866606	1.65748	1.868857	1.643791	1.911129
1.400085	1.390996	1.395	1.401176	1.38457	1.37275
1.40262	1.463164	1.389325	1.463503	1.381245	1.474585
0.001497	0.00204	0.005783	0.001734	-0.0093	0.001458
-1.400664	-1.458877	-1.379493	-1.461485	-1.394595	-1.469527
-1.400523	-1.391806	-1.386366	-1.401715	-1.393281	-1.363794
-0.00088	-0.000636	0.003183	-0.001062	-0.004597	0.003093
2.276323	2.344484	2.28995	2.365533	2.285496	2.340299
2.792751	2.779431	2.804175	2.800561	2.804177	2.760537
2.279834	2.279295	2.290313	2.284713	2.281277	2.274705
2.27978	2.280906	2.28014	2.279342	2.282767	2.281805
2.793275	2.775958	2.758173	2.771117	2.804987	2.79737
2.276227	2.347972	2.279393	2.335645	2.287455	2.386159
-2.278191	-2.346438	-2.276793	-2.366815	-2.292007	-2.340251
-2.792743	-2.779213	-2.752327	-2.800552	-2.829138	-2.760368
-2.277357	-2.277346	-2.272295	-2.281747	-2.297094	-2.274053
-2.277425	-2.278061	-2.283061	-2.277381	-2.295714	-2.281442
-2.793227	-2.776279	-2.79895	-2.770743	-2.825919	-2.791567
-2.278419	-2.351404	-2.286177	-2.337377	-2.291565	-2.378595
-2.786988	-2.863496	-2.776099	-2.879039	-2.787078	-2.86565
2.787362	2.862264	2.781856	2.879121	2.779548	2.869437

Table S6. Hirshfeld atomic charge of An&B_{40} and An@B_{40} (Heavy elements in bold)

NpB_{40}		PuB_{40}		UB_{40}	
Endohedral	Exohedral	Endohedral	Exohedral	Endohedral	Exohedral
**0.07291**	**-0.000091**	**-0.033497**	**0.005638**	**0.001595**	**0.000999**
-1.683347	-1.89975	-1.712934	-1.875282	-1.705205	-1.904503
-1.628128	-1.679137	-1.671234	-1.677524	-1.641875	-1.681442
-1.414825	-1.458221	-1.453124	-1.442157	-1.450671	-1.465828
1.668231	1.778177	1.659066	1.774836	1.70163	1.77793
-0.005412	-0.000062	-0.024162	0.000582	0.000267	0.000413
-2.63364	-2.63915	-2.64586	-2.634913	-2.662501	-2.643988
2.348049	2.39304	2.302055	2.393671	2.365266	2.38898
2.340742	2.382867	2.325254	2.376473	2.403342	2.385623
1.671958	1.900383	1.658053	1.882708	1.70492	1.906856
1.642586	1.679251	1.606065	1.680207	1.641692	1.681967
1.434651	1.458718	1.390628	1.449313	1.452537	1.461808
-2.675583	-2.749869	-2.708071	-2.749845	-2.743382	-2.75429
-1.416053	-1.397474	-1.452484	-1.403446	-1.451633	-1.399282
1.437452	1.397017	1.390705	1.403208	1.452306	1.39929
0.845224	0.881098	0.834233	0.881638	0.867045	0.882232
-0.870606	-0.850949	-0.886199	-0.850876	-0.867005	-0.852632
2.341952	2.373637	2.301891	2.368808	2.365914	2.378206
-0.872714	-0.880994	-0.886583	-0.88232	-0.86762	-0.881426
0.004213	-0.000121	-0.031303	-0.000373	0.000015	-0.000062
-2.669572	-2.825621	-2.708367	-2.80289	-2.744408	-2.830437
-2.306693	-2.373292	-2.36545	-2.364118	-2.365742	-2.37763
-2.319976	-2.392908	-2.364681	-2.390738	-2.364408	-2.389861
2.33894	2.372539	2.325833	2.374284	2.40621	2.377216
-2.354038	-2.382543	-2.378701	-2.370532	-2.400858	-2.38583
-2.356483	-2.372579	-2.378807	-2.372997	-2.406795	-2.377035
0.874941	0.882577	0.838391	0.884199	0.870003	0.884199
-0.863109	-0.882543	-0.903324	-0.883324	-0.869854	-0.884309
-1.680981	-1.778344	-1.712736	-1.775438	-1.702147	-1.778137
-1.393057	-1.378697	-1.405159	-1.378503	-1.395691	-1.384833
2.59922	2.63928	2.594628	2.638796	2.661377	2.643922
0.848114	0.851749	0.834523	0.858512	0.865871	0.852801
0.874134	0.853226	0.837977	0.855647	0.870469	0.852921
-0.861557	-0.852711	-0.903231	-0.849673	-0.870135	-0.853126
-1.39188	-1.411426	-1.40515	-1.406066	-1.397387	-1.410008
2.671615	2.749749	2.651154	2.749865	2.744223	2.753516
0.005583	0.000237	-0.031522	0.003721	0.000622	-0.001129
1.373833	1.378509	1.355729	1.379697	1.396318	1.386946
-0.008435	0.000179	-0.024285	0.003304	-0.001473	0.000544
2.664799	2.825934	2.650185	2.80936	2.744316	2.831261
1.361812	1.411758	1.355917	1.411394	1.396676	1.409987

Table S7. *f* orbital information of the U&B*m* and U@B*n* (m, n = 36, 38, 40) (M is Mulliken and E is Loewdin)

	B36						B38						B40					
	atom	Endohedral (M)	Endohedral (E)	atom	Exohedral (M)	Exohedral (E)	atom	Endohedral (M)	Endohedral (E)	atom	Exohedral (M)	Exohedral (E)	atom	Endohedral (M)	Endohedral (E)	atom	Exohedral (M)	Exohedral (E)
charge																		
f	16.99971	18.26761	17.84086	17.00058	16.52336	16.82526	17.99438	18.65474	18.05631	17.99396	17.07845	17.40426	17.00009	18.06842	18.01976	18.00015	17.06910	17.40808
f0	2.35197	2.92254	2.91792	2.68326	2.45384	2.49077	2.30317	3.38172	3.34528	2.78321	2.36816	2.40526	2.17999	2.47178	2.46059	2.95430	2.63658	2.68084
f+1	2.21041	2.66138	2.59969	2.15971	2.94185	2.99074	2.97448	2.67056	2.53204	2.77438	2.48424	2.50396	2.75274	2.19972	2.20336	2.34984	2.27295	2.29649
f-1	2.78744	2.33337	2.28766	2.32965	2.01004	2.03473	2.54047	2.38868	2.33080	2.19732	2.48040	2.52950	2.43122	2.37820	2.37005	2.26088	2.29933	2.35020
f+2	2.64624	2.57715	2.48835	2.44002	2.67083	2.69889	2.28304	2.54104	2.44512	2.39426	2.07602	2.12230	2.20174	2.26730	2.26613	2.78591	2.64268	2.68122
f-2	2.10892	2.63097	2.46736	2.57156	2.17252	2.24050	2.77622	2.58193	2.44274	2.71248	2.76919	2.83227	2.54183	4.07938	4.03809	2.57962	2.30522	2.39342
f+3	2.86191	2.88623	2.85209	2.57776	2.11882	2.15031	2.90325	2.45065	2.35603	2.72269	2.62165	2.67661	2.84798	2.12927	2.14105	2.91324	2.68984	2.72842
f-3	2.03282	2.25598	2.22779	2.23862	2.15546	2.21934	2.21376	2.64015	2.60430	2.40961	2.27880	2.33436	2.04460	2.54278	2.54050	2.15635	2.22249	2.27749
spin																		
f	2.99871	2.09237	1.98994	2.99935	2.09390	2.06682	3.99428	none	none	3.99372	2.79499	2.75807	2.99892	none	none	3.99991	2.81972	2.78435
f0	0.35151	0.81244	0.78704	0.68314	0.39461	0.39164	0.30316			0.78317	0.34574	0.34237	0.17991			0.95426	0.60133	0.59360
f+1	0.21035	0.35283	0.33483	0.15959	0.86921	0.85874	0.97446			0.77437	0.47176	0.46669	0.75266			0.34983	0.26695	0.26645
f-1	0.78731	0.02359	0.01618	0.32954	0.00178	0.00175	0.54047			0.19732	0.45111	0.44652	0.43116			0.26088	0.24495	0.24224
f+2	0.64624	0.14644	0.13544	0.43967	0.61644	0.61235	0.28304			0.39424	0.05887	0.05720	0.20168			0.78582	0.61605	0.60892
f-2	0.10887	0.09228	0.07501	0.57152	0.06388	0.05660	0.77621			0.71237	0.69988	0.69281	0.54149			0.57962	0.27776	0.27347
f+3	0.86177	0.64627	0.62775	0.57753	0.07715	0.07547	0.90321			0.72265	0.54833	0.53541	0.84764			0.91314	0.65040	0.64111
f-3	0.03267	0.01852	0.01370	0.23837	0.07083	0.07027	0.21375			0.40960	0.21932	0.21708	0.04439			0.15635	0.16227	0.15858

Table S8. *f* orbital information of the Pu&B*m* and Pu@B*n* (m, n = 36, 38, 40) (M is Mulliken and E is Loewdin)

	B36						B38						B40					
	atom	Endohedral (M)	Endohedral (E)	atom	Exohedral (M)	Exohedral (E)	atom	Endohedral (M)	Endohedral (E)	atom	Exohedral (M)	Exohedral (E)	atom	Endohedral (M)	Endohedral (E)	atom	Exohedral (M)	Exohedral (E)
charge																		
f	20.00069	20.11168	19.93101	20.00067	19.12244	19.39853	20.00099	20.64270	20.47525	20.00086	19.15839	19.43114	20.00050	19.88117	20.08486	20.00089	19.17194	19.46940
f0	2.99638	3.06660	3.07321	2.96203	2.52919	2.56178	2.73796	3.12390	3.13050	2.95043	2.34949	2.38688	2.84723	2.58378	2.60504	2.89399	2.86700	2.90382
f+1	2.84521	2.76633	2.74177	2.80946	3.00170	3.05061	2.88948	2.72872	2.68706	2.67020	2.90923	2.91940	2.98328	2.87908	2.90211	2.95983	2.65718	2.67639
f-1	2.99125	2.67281	2.64900	2.98772	2.39119	2.40796	2.99789	3.13060	3.11614	2.83637	2.95221	2.98684	2.91846	2.83680	2.87758	2.98500	2.83297	2.87826
f+2	2.97050	2.56246	2.51840	2.86294	2.59292	2.62365	2.67615	3.19575	3.16108	2.65329	2.88636	2.92255	2.54562	3.10833	3.13867	2.50723	2.77760	2.81148
f-2	2.69822	3.31544	3.24427	2.62771	2.97508	3.03887	2.87527	2.38884	2.33637	2.92144	2.90809	2.96767	2.90128	2.76051	2.78928	2.99914	2.50137	2.58161
f+3	2.99967	2.82175	2.81024	2.95785	3.00588	3.03088	2.95066	2.93619	2.90557	2.99893	3.01440	3.06202	2.99794	2.82006	2.84993	2.66196	2.98332	3.02001
f-3	2.49947	2.90629	2.89413	2.79297	2.62649	2.68479	2.87358	3.13869	3.13852	2.97021	2.13862	2.18579	2.80669	2.89261	2.92226	2.99374	2.55249	2.59784
spin																		
f	5.99813	4.49778	4.38344	5.99883	4.91675	4.88147	5.99814	4.64691	4.53820	5.99836	4.90369	4.86144	5.99913	4.93495	4.87503	5.99861	4.94416	4.90676
f0	0.99584	0.97661	0.96228	0.96172	0.50073	0.49860	0.73777	0.83186	0.81081	0.95029	0.32949	0.32636	0.84704	0.40735	0.39871	0.89354	0.83591	0.82753
f+1	0.84468	0.54204	0.52188	0.80925	0.95867	0.95213	0.88890	0.35282	0.33569	0.67007	0.90044	0.89573	0.98310	0.76302	0.75545	0.95944	0.65233	0.65011
f-1	0.99087	0.43112	0.41258	0.98739	0.38757	0.38522	0.99776	0.91416	0.90344	0.83546	0.92828	0.92160	0.91845	0.73697	0.73087	0.98492	0.78491	0.78068
f+2	0.97006	0.24575	0.22145	0.86248	0.56236	0.55949	0.67561	0.91786	0.90562	0.65284	0.87303	0.86685	0.54535	0.96677	0.96015	0.50641	0.75189	0.74640
f-2	0.69773	0.92605	0.92357	0.62734	0.93367	0.92404	0.87481	0.06461	0.04783	0.92111	0.84322	0.83547	0.90095	0.59878	0.58973	0.99890	0.47586	0.47150
f+3	0.99963	0.63756	0.62094	0.95782	0.98026	0.97509	0.95015	0.68015	0.66986	0.99857	0.95536	0.94414	0.99771	0.74114	0.73397	0.66174	0.94883	0.94076
f-3	0.49933	0.73867	0.72073	0.79283	0.59349	0.58691	0.87314	0.88546	0.86496	0.97002	0.07387	0.07129	0.80654	0.72093	0.70615	0.99368	0.49444	0.48977

Table S9. *f* orbital information of the Np&B*m* and Np@B*n* (m, n = 36, 38, 40) (M is Mulliken and E is Loewdin)

	B36						B38						B40					
	atom	Endohedral (M)	Endohedral (E)	atom	Exohedral (M)	Exohedral (E)	atom	Endohedral (M)	Endohedral (E)	atom	Exohedral (M)	Exohedral (E)	atom	Endohedral (M)	Endohedral (E)	atom	Exohedral (M)	Exohedral (E)
charge																		
f	19.00039	19.27523	18.96141	19.00079	18.09604	18.38411	19.00038	19.59351	19.23638	19.00056	18.12182	18.40830	18.98536	18.81428	19.05617	18.47955	18.08601	18.40480
f0	2.84748	2.98038	2.97715	2.30306	2.89022	2.91543	2.86120	3.22979	3.22370	2.66345	2.44097	2.48093	2.56970	2.27634	2.30165	2.89372	2.26708	2.30946
f+1	2.80631	2.92052	2.88071	2.91865	2.39726	2.45285	2.74170	2.60594	2.52342	2.83799	2.48209	2.49076	2.75795	3.03745	3.04616	2.58486	3.09084	3.10942
f-1	2.62355	2.44442	2.40854	2.66463	2.43654	2.45556	2.28621	2.36746	2.32864	2.93371	2.89692	2.93163	2.67864	2.97823	3.03932	2.33787	2.52126	2.56926
f+2	2.35434	2.50383	2.44516	2.59207	2.92732	2.95433	2.94445	3.25305	3.19236	2.79003	2.26844	2.31085	2.64778	2.39907	2.42737	2.89100	2.31150	2.35175
f-2	2.82731	2.82272	2.69453	2.57881	2.20777	2.27939	2.85578	2.49979	2.40617	2.57392	2.83196	2.89642	2.95753	2.61457	2.66890	2.45005	2.59894	2.68133
f+3	2.73842	2.56363	2.54160	2.96229	2.40098	2.42992	2.94611	3.19296	3.13640	2.25375	2.80156	2.84847	2.99693	2.69558	2.72797	2.95514	3.21457	3.24891
f-3	2.80299	3.03972	3.01371	2.98129	2.83596	2.89663	2.36494	2.44452	2.42570	2.94770	2.39989	2.44923	2.37683	2.81305	2.84479	2.36691	2.08184	2.13468
spin																		
f	4.99974	3.43712	3.29446	4.99868	3.87930	3.82987	4.99977	2.87357	2.80284	4.99838	3.87816	3.84478	4.98467	3.83808	3.76653	4.47800	2.00955	1.98755
f0	0.84742	0.87771	0.85587	0.30245	0.86155	0.84692	0.86115	0.76977	0.76093	0.66322	0.41971	0.41575	0.56965	0.10134	0.09328	0.89364	0.24274	0.24260
f+1	0.80628	0.65926	0.64040	0.91822	0.35615	0.34854	0.74161	0.13002	0.11400	0.83780	0.47587	0.47390	0.75789	0.90456	0.89117	0.58468	0.73802	0.73492
f-1	0.62339	0.17461	0.15915	0.66396	0.43264	0.42979	0.28618	0.05959	0.05233	0.93362	0.87167	0.86540	0.67859	0.89192	0.88140	0.33753	0.47763	0.46952
f+2	0.35424	0.14046	0.12611	0.59203	0.89388	0.88714	0.94439	0.88449	0.87888	0.78996	0.25580	0.25305	0.64758	0.24322	0.23704	0.89090	0.27437	0.27197
f-2	0.82710	0.38263	0.34852	0.57875	0.16175	0.15548	0.85564	0.07521	0.06035	0.57327	0.76030	0.75428	0.95746	0.46742	0.45967	0.44975	-0.14311	-0.14518
f+3	0.73837	0.35893	0.34159	0.96205	0.37384	0.36885	0.94590	0.86785	0.86284	0.25318	0.76187	0.75269	0.99671	0.60811	0.60019	0.95505	0.40328	0.39827
f-3	0.80294	0.84354	0.82282	0.98123	0.79950	0.79315	0.36490	0.08663	0.07351	0.94733	0.33296	0.32972	0.37679	0.62151	0.60377	0.36646	0.01662	0.01545

Table S10. The information of dipole moment for Endohedral Borospherenes

		Dipole moment (Debye)			Rotational constants (cm^{-1})			Single point energy (Eh)
		x	y	z	x	y	z	
$Np@B_{36}$	CPCM (water)	0.00171	0.00269	0.00284	0.007486	0.007486	0.007485	-31177.61164
	vacuum	0.00016	-0.00032	0.00043	0.007487	0.007486	0.007486	-31177.60856
$Np@B_{38}$	CPCM (water)	0.00248	0.00694	0.00176	0.007472	0.00694	0.006903	-31227.17204
	vacuum	0.00049	0.00194	-0.00115	0.007464	0.006944	0.006902	-31227.16783
$Np@B_{40}$	CPCM (water)	-0.08972	0.00491	0.06661	0.006164	0.00576	0.005665	-31276.77846
	vacuum	-0.03381	-0.00327	0.01447	0.006164	0.00576	0.005665	-31276.77524
$Pu@B_{36}$	CPCM (water)	0.00098	-0.00031	0.00001	0.007479	0.007479	0.007421	-32067.29224
	vacuum							
$Pu@B_{38}$	CPCM (water)	0.00302	0.01279	0.00269	0.007434	0.006948	0.006902	-32116.85125
	vacuum	0.00185	0.00409	-0.00133	0.007435	0.00695	0.006897	-32116.84682
$Pu@B_{40}$	CPCM (water)	0.00741	-0.00258	0.03688	0.006127	0.005784	0.005683	-32166.48886
	vacuum	0.00109	-0.001	0.00302	0.006131	0.005781	0.005681	-32166.48572
$U@B_{36}$	CPCM (water)	0.01772	0.00037	0.00416	0.007512	0.007502	0.007407	-30307.44561
	vacuum	0.00162	-0.0006	0.00349	0.007512	0.007505	0.007406	-30307.44271
$U@B_{38}$	CPCM (water)	0.88966	-0.0114	-0.02014	0.007512	0.006924	0.006901	-30357.01708
	vacuum	0.10488	0.00173	0.00357	0.007515	0.006939	0.006913	-30357.01223
$U@B_{40}$	CPCM (water)	0.00281	0.02754	0.00039	0.006037	0.005889	0.005887	-30406.62207
	vacuum	-0.00158	0.01692	0.00192	0.006039	0.005889	0.005886	-30406.61837

Table S11. The information of dipole moment for Exohedral Borospherenes

		Dipole moment (Debye)			Rotational constants (cm^{-1})			Single point energy (Eh)
		x	y	z	x	y	z	
$Np\&B_{36}$	CPCM (water)	-11.69427	-0.02647	-0.02255	0.007601	0.003259	0.003256	-31177.44992
	vacuum	-4.32896	-0.03945	-0.00318	0.007589	0.00333	0.003329	-31177.39356
$Np\&B_{38}$	CPCM (water)	0.00314	10.19123	-0.02874	0.006895	0.003293	0.003134	-31227.0741
	vacuum	-0.00592	4.0625	-0.07394	0.006889	0.003358	0.003193	-31227.02622
$Np\&B_{40}$	CPCM (water)	0.01171	8.62912	0.70068	0.005882	0.002908	0.002893	-31276.71391
	vacuum	-0.0001	3.42163	0.31894	0.005869	0.002951	0.002936	-31276.67379
$Pu\&B_{36}$	CPCM (water)	-11.21012	-0.09102	-0.00979	0.007554	0.003214	0.003209	-32067.15319
	vacuum	-4.3397	-0.03944	0.00483	0.007557	0.003298	0.003288	-32067.09973
$Pu\&B_{38}$	CPCM (water)	0.01064	9.98212	0.01813	0.006881	0.003252	0.003095	-32116.77698
	vacuum	0.00007	4.05288	-0.1313	0.006878	0.003327	0.00316	-32116.73098
$Pu\&B_{40}$	CPCM (water)	0.0118	9.23772	0.40001	0.005851	0.002869	0.002842	-32166.41648
	vacuum	0.00315	3.41186	0.31962	0.005859	0.002919	0.002895	-32166.37781
$U\&B_{36}$	CPCM (water)	-12.03512	0.01444	-0.0093	0.007447	0.003353	0.003326	-30307.27473
	vacuum	-4.59686	0.02873	-0.03619	0.007514	0.003383	0.003375	-30307.20975
$U\&B_{38}$	CPCM (water)	-0.08892	10.34458	-0.40235	0.006898	0.003319	0.003151	-30356.8847
	vacuum							
$U\&B_{40}$	CPCM (water)	-0.00216	9.18959	0.73021	0.00588	0.002918	0.002904	-30406.52636
	vacuum	-0.00716	3.56546	0.26942	0.005861	0.002956	0.002943	-30406.48065

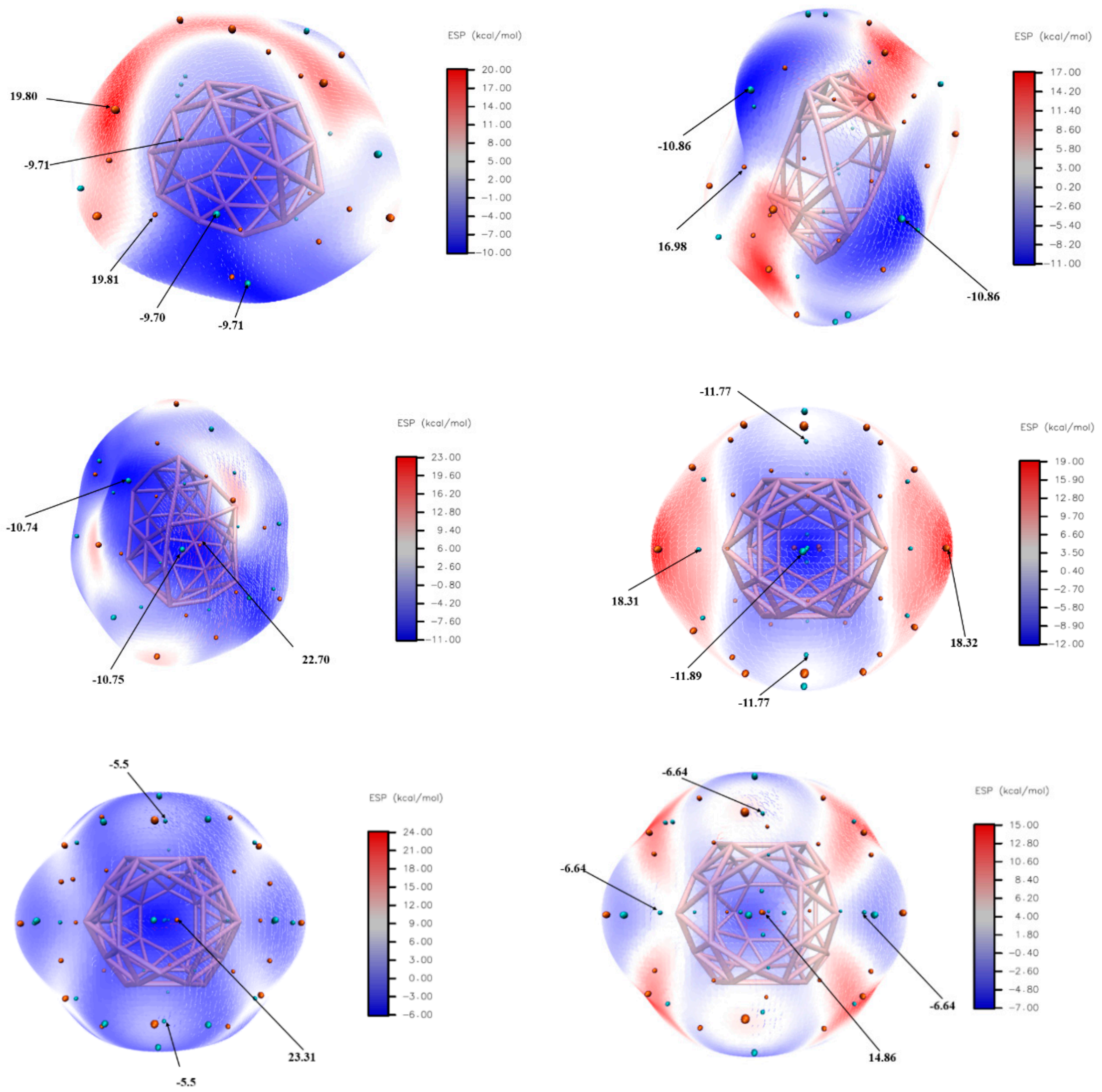

Figure S1. ESP-mapped molecular vdW surfaces of B_m (m = 28, 32, 34, 36, 38, 40) at the PBE-def2- TZVPP level.

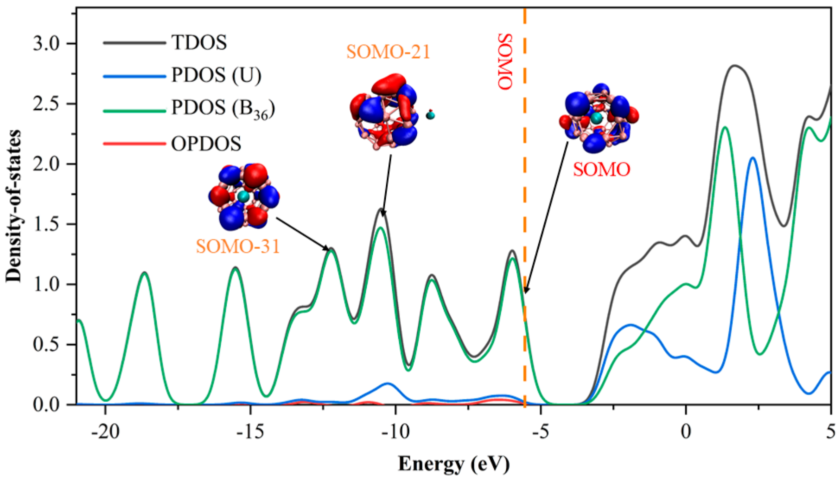

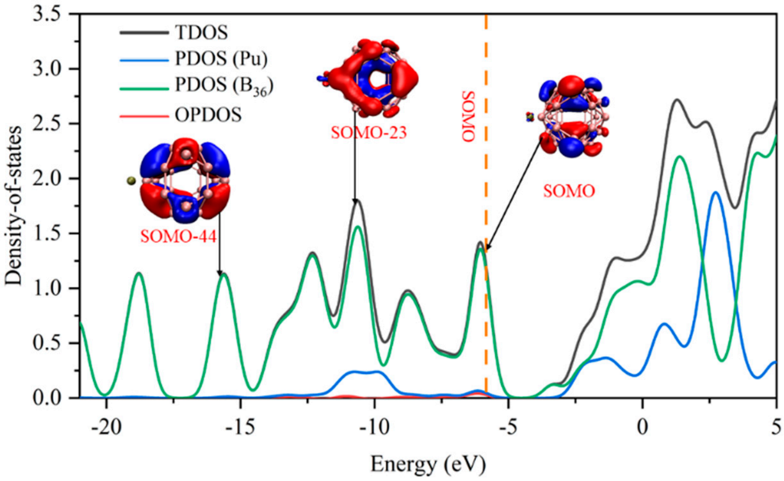

Figure S2. The TDOS, PDOS and OPDOS curves of U&B_{36} and Pu&B_{36} at the PWPB95-ZORA/def2-TZVPP-SARC level.

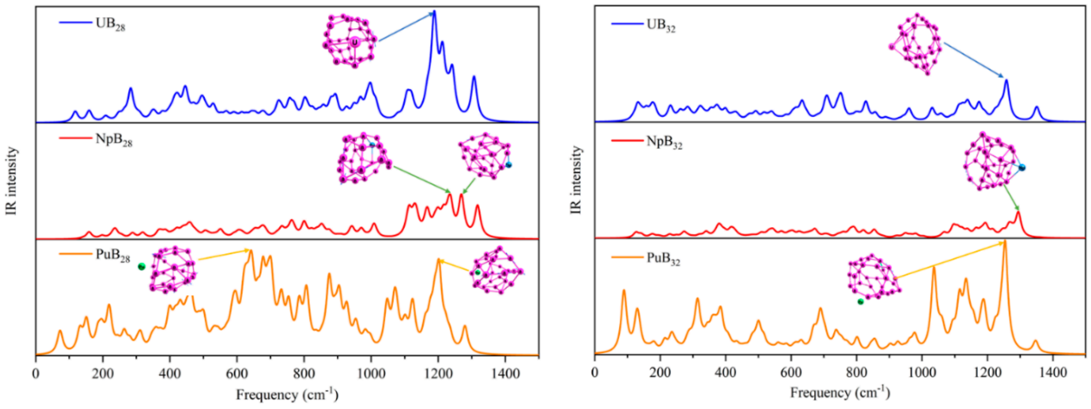

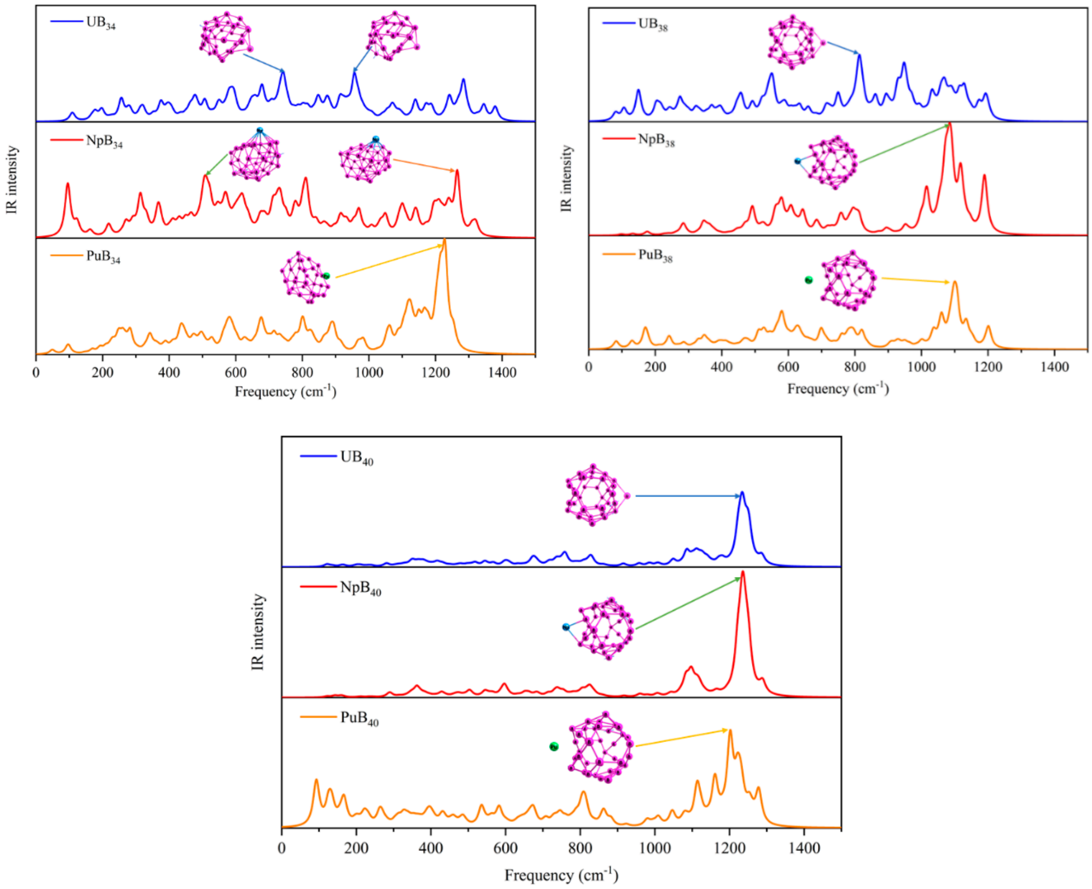

Figure S3. IR spectra of An&B_m (m = 28, 32, 34, 38, 40).

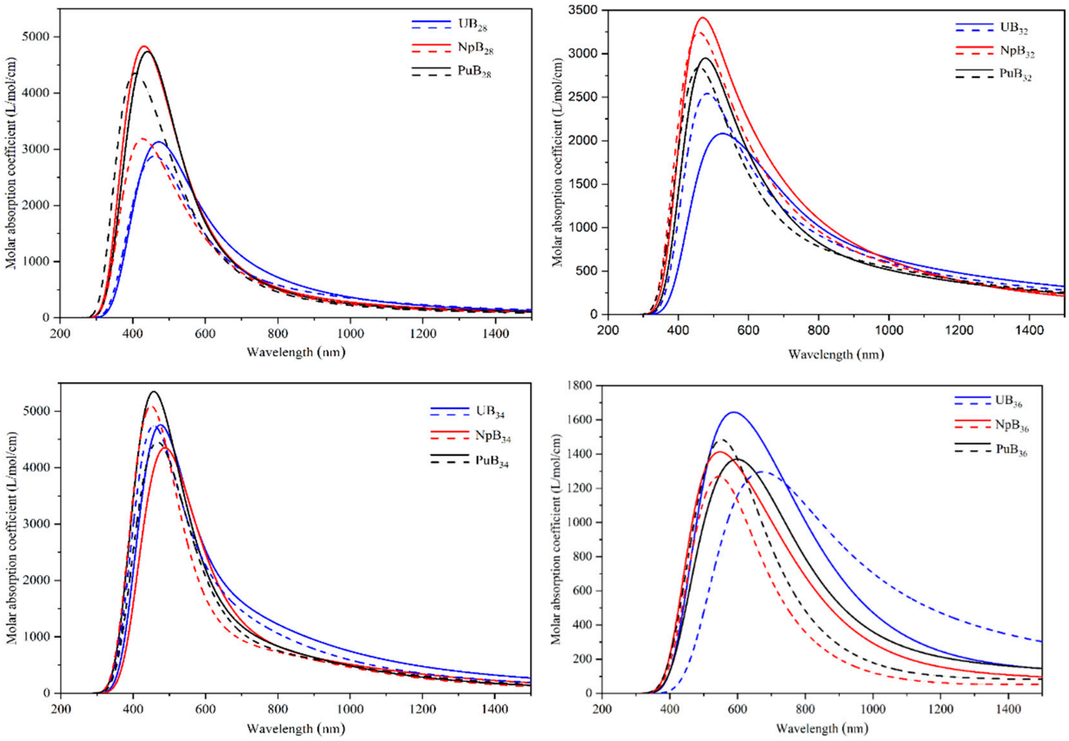

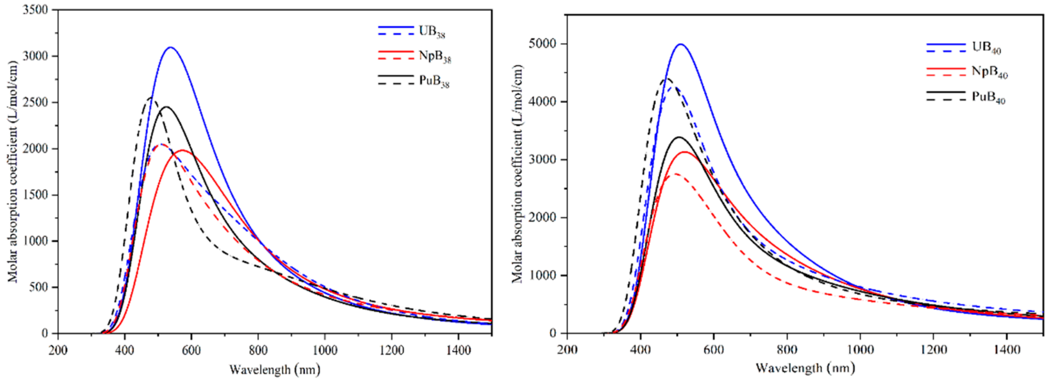

Figure S4. UV/Vis spectra of the An&B_m (An = U, Np, Pu; m=28, 32, 34,36, 38, 40) in vacuum (solid lines) and in water using the CPCM (dash lines) at the PBE0-ZORA/def2-TZVPP-SARC level.

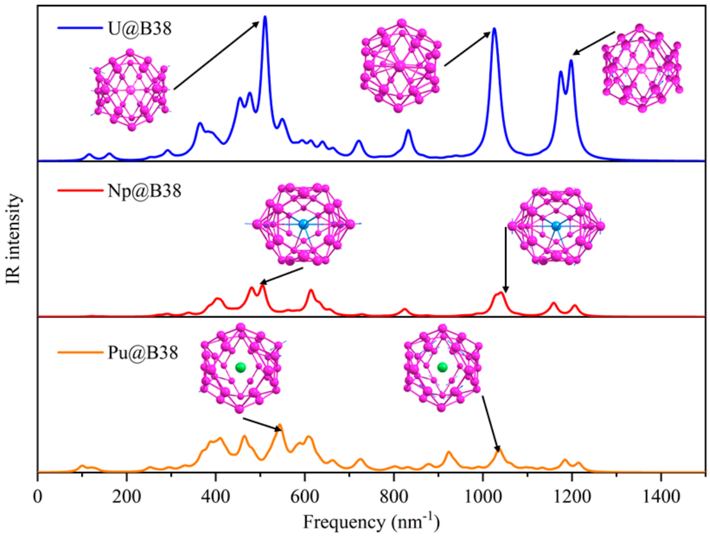

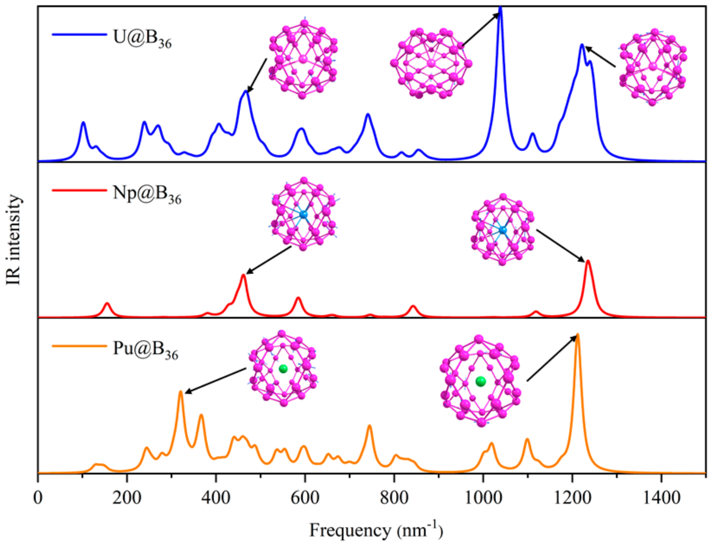

Figure S5: IR spectra of An@B_n (An = U, Np, Pu; n = 36, 38).

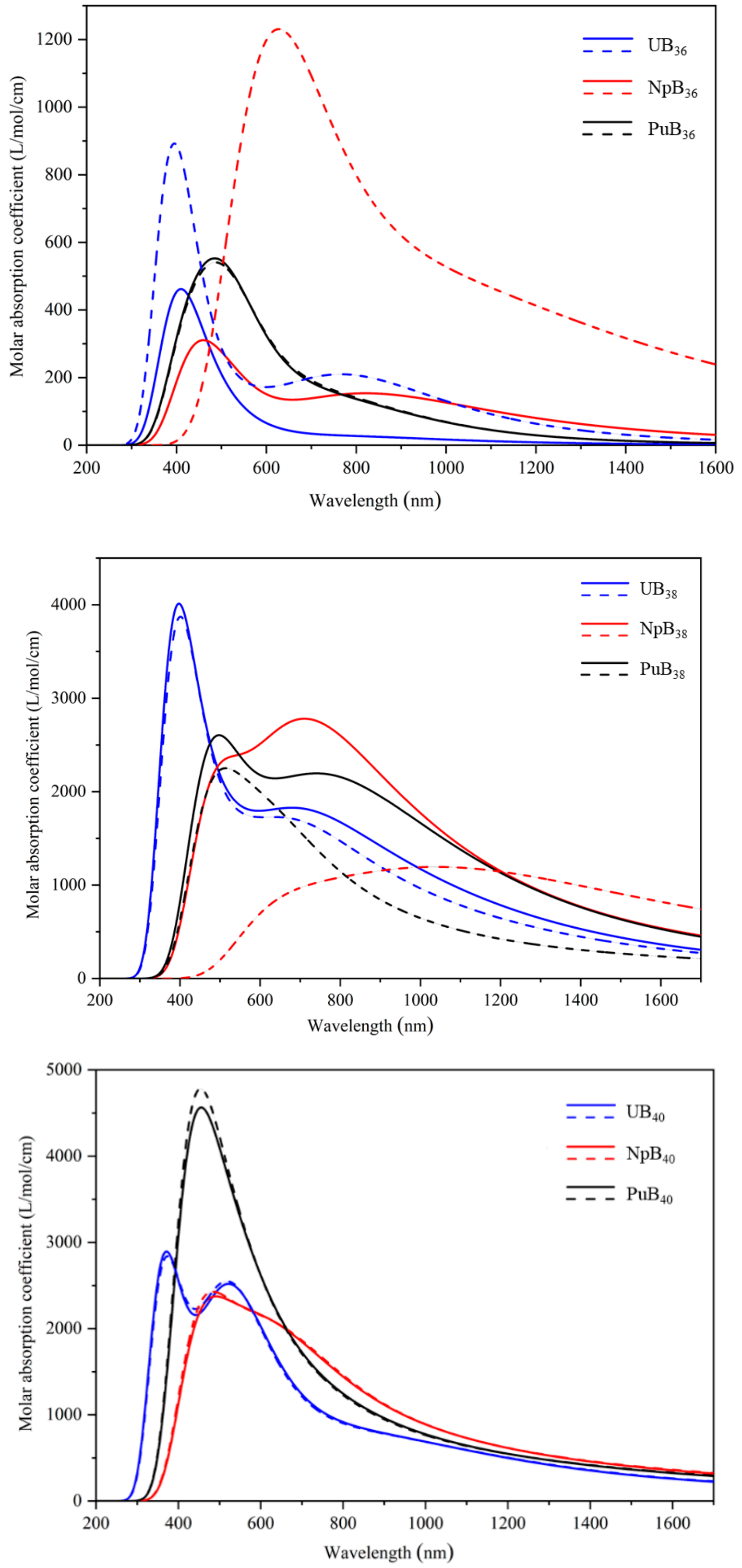

Figure S6. UV/Vis spectra of the An@B_n (An = U, Np, Pu; n= 36, 38, 40) in vacuum (solid lines) and in water using the CPCM (dash lines) at the PBE0-ZORA/def2-TZVPP-SARC level.

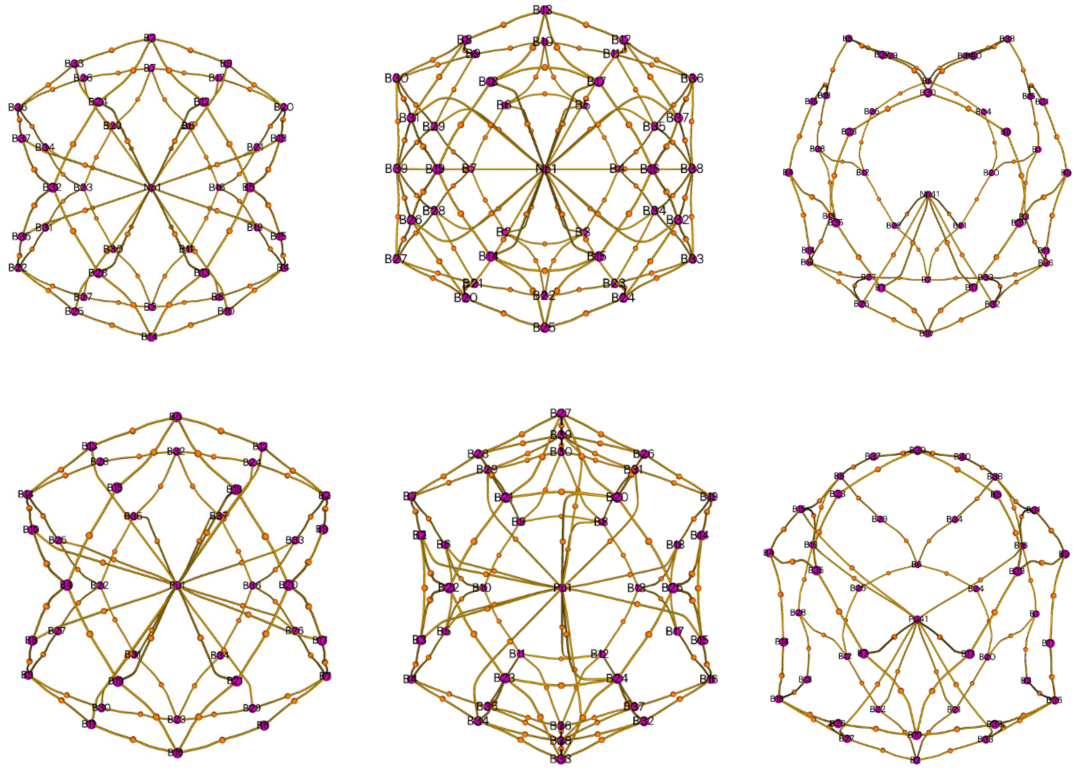

Figure S7. QTAIM molecular graph of Np@B_n and Pu@B_n (n=36，38，40). Red points represent bond critical points, lines represent bond paths.

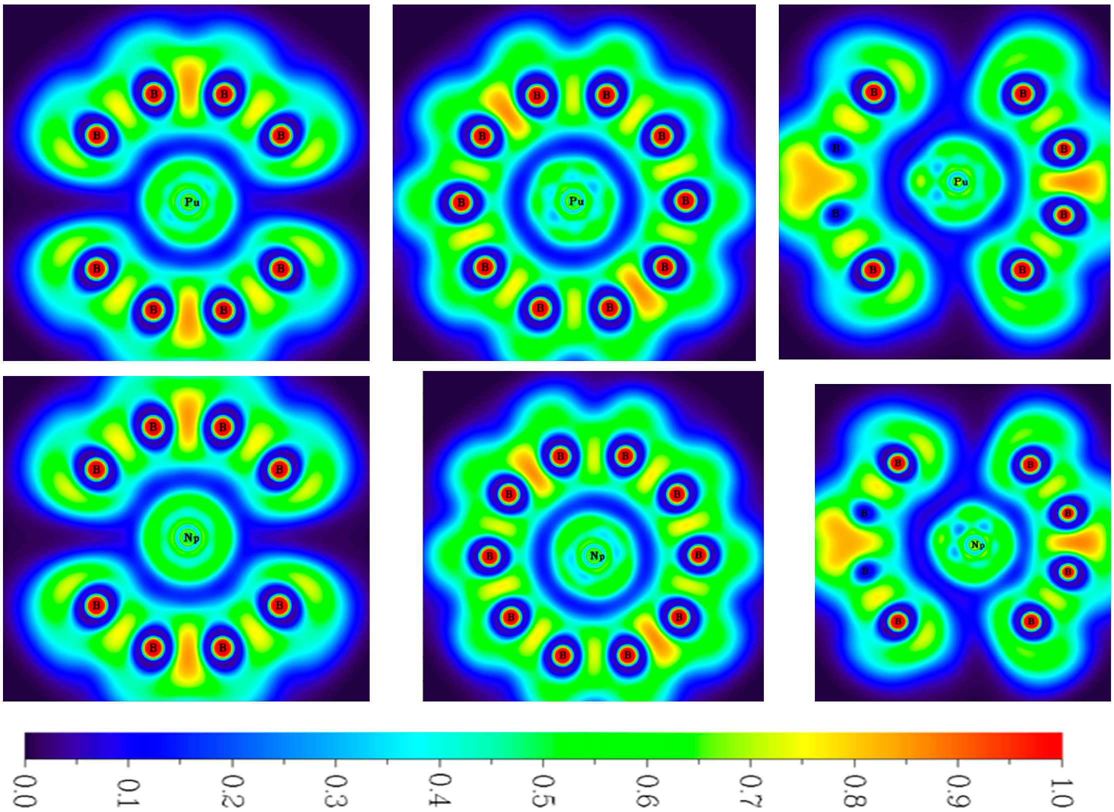

Figure S8: Two-dimensional color-filled plane map of ELF for Np@B_n and Pu@B_n (n = 36, 38, 40) at the PBE-ZORA/def2-SVP-SARC level.

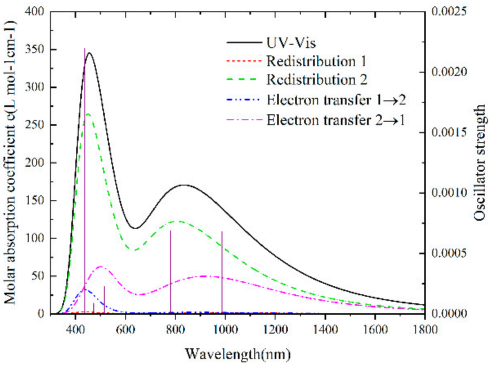

NpB_{36}

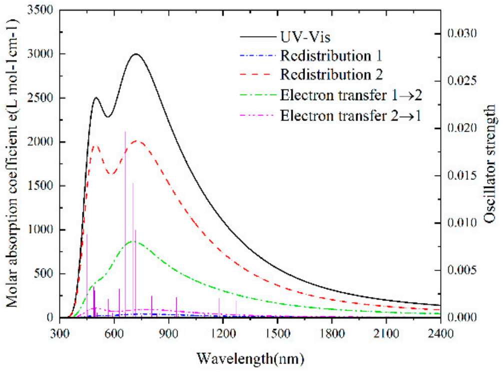

NpB_{38}

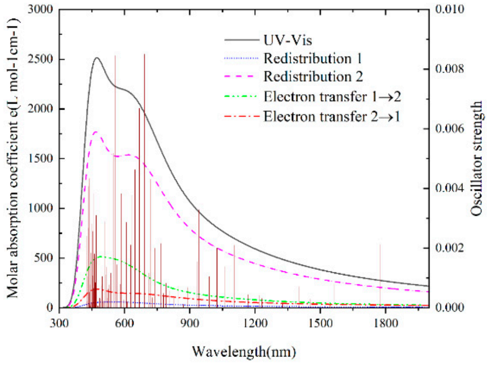

NpB_{40}

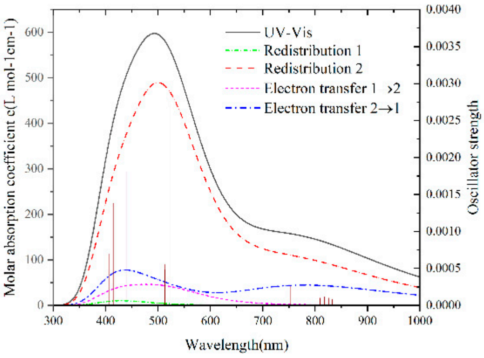

PuB_{36}

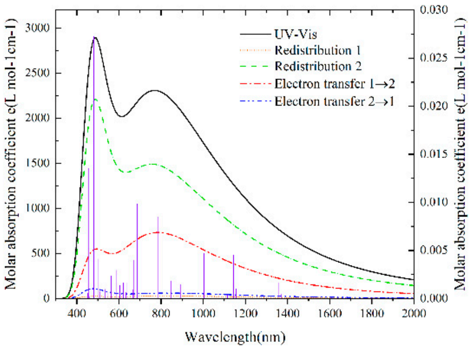

PuB_{38}

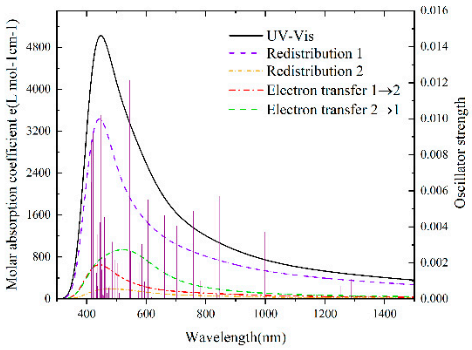

PuB_{40}

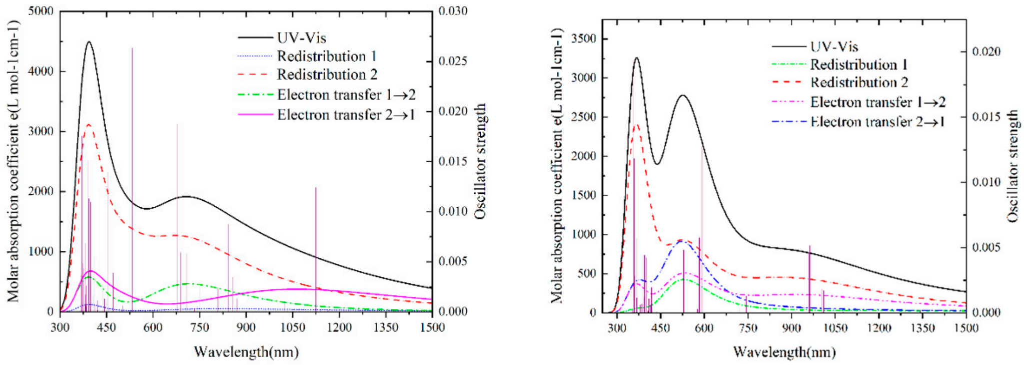

UB_{36} UB_{38}

Figure S9. Charge-transfer spectrum (CTS) of An@B_n (An = U, Np, Pu; n = 36, 38, 40) at PBE0 ZORA/def2-TZVPP-SARC level.
